# Cost-effectiveness of novel diagnostic tools for idiopathic pulmonary fibrosis in the United States

**DOI:** 10.1186/s12913-025-12506-1

**Published:** 2025-03-15

**Authors:** Christopher J. Cadham, Joshua Reicher, Michael Muelly, David W. Hutton

**Affiliations:** 1 Department of Health Management and Policy, School of Public Health, University of Michigan, 1420 Washington Heights, Ann Arbor, MI 48109-2013 USA; 2 Imvaria, Inc, Berkeley, CA USA

**Keywords:** Cost-effectiveness analysis, Idiopathic pulmonary fibrosis, Machine learning/classification

## Abstract

**Objectives:**

Novel non-invasive machine learning algorithms may improve accuracy and reduce the need for biopsy when diagnosing idiopathic pulmonary fibrosis (IPF). We conducted a cost-effectiveness analysis of diagnostic strategies for IPF.

**Methods:**

We developed a decision analytic model to evaluate diagnostic strategies for IPF in the United States. To assess the full spectrum of costs and benefits, we compared four interventions: a machine learning diagnostic algorithm, a genomic classifier, a biopsy-all strategy, and a treat-all strategy. The analysis was conducted from the health sector perspective with a lifetime horizon. The primary outcome measures were costs, Quality-Adjusted Life-Years (QALYs) gained, and Incremental Cost-Effectiveness Ratios (ICERs) based on the average of 10,000 probabilistic runs of the model.

**Results:**

Compared to a biopsy-all strategy the machine learning algorithm and genomic classifer reduced diagnostic-related costs by $14,876 and $3,884, respectively. Use of the machine learning algorithm consistently reduced diagnostic costs. When including downstream treatment costs and benefits of anti-fibrotic treatment, the machine learning algorithm had an ICER of $331,069 per QALY gained compared to the biopsy-all strategy. The genomic classifier had a higher ICER of $390,043 per QALY gained, while the treat-all strategy had the highest ICER of $3,245,403 per QALY gained. Results were sensitive to changes in various input parameters including IPF treatment costs, sensitivity and specificity of novel screening tools, and the rate of additional diagnostics following inconclusive results. High treatment costs were found to drive overall cost regardless of the diagnostic method. As treatment costs lowered, the supplemental diagnostic tools became increasingly cost-effective.

**Conclusions:**

Novel tools for diagnosing IPF reduced diagnostic costs, while overall incremental cost-effectiveness ratios were high due to treatment costs. New IPF diagnosis approaches may become more favourable with lower-cost treatments for IPF.

**Supplementary Information:**

The online version contains supplementary material available at 10.1186/s12913-025-12506-1.

## Introduction

Idiopathic Pulmonary Fibrosis (IPF) is the most common Interstitial Lung Disease (ILD), a category of relatively uncommon, chronic lung diseases which lead to substantial morbidity and mortality due to progressive lung function impairment [1, 2]. IPF occurs primarily in older adults, particularly over 75 [3], and is more common among men and people who smoke cigarettes [2]. IPF is caused by the interaction of multiple environmental and genetic factors, and repetitive local micro-injuries to aging lung tissue with no singular cause identified [2, 4]. While the disease trajectory varies for patients, the prognosis is generally poor, with high morbidity and a median survival of 3.8 years [5]. Therapeutics have come to market in the past decade, which slow the decline in lung function and reduce respiratory events, such as acute exacerbations [6–9]. Pooled analyses suggest there may be improvements to survival with long-term treatment [10–13]. However, these medications come with high costs relative to their benefits [14, 15]. Given their high cost and the still developing evidence of the benefits of treatment on survival, the accurate and early diagnosis of IPF is essential to ensuring the maintenance of quality of life.

Current guidelines for diagnosing IPF recommend the collection of detailed clinical history to rule out causes of other ILD (e.g. chronic exposure to birds associated with hypersensitivity pneumonitis), pulmonary function testing, a variety of blood-based inflammatory biomarkers, and chest imaging with high-resolution computed tomography [16, 17]. Multidisciplinary discussion (MDD) is used to determine whether or not a supplemental assessment is necessary. The overlap between ILD subtypes is significant and subjective interpretation of imaging leads to diagnostic disagreement among clinicians and even subspecialized pulmonologists [18]. Such disagreement can exacerbate existing delays in referral to specialist care [19]. Recommended supplemental diagnostics includes either surgical lung biopsy or bronchoalveolar lavage, with surgical lung biopsy representing a key element of the current gold standard for diagnosis. Surgical lung biopsies are costly [20], come with a severe risk of death and complications [21] and have some limitations to the accuracy of histological confirmation [22]. 

Recently, novel screening tools have emerged for the diagnosis of IPF that avoid high cost and potentially life threatening surgical lung biopsy. A pathology-based genomics classification system was developed [23, 24]. However, it still requires an invasive transbronchial biopsy with collection of at least 4–5 tissue samples and can result in mixed effects [23]. Machine learning tools which analyze medical images also present another opportunity for non-invasive diagnostics [25, 26]. In pulmonary care, machine learning has been beneficial in interpreting chronic obstructive pulmonary disease and pulmonary function testing [27]. Initial research suggests that such algorithms can aid in the assessment of ILD cases, although their impact on survival is unknown [28]. 

The adoption of non-invasive diagnostic algorithms may provide multiple potential benefits in the diagnosis of IPF. Among those cases that an MDD would refer to a biopsy, a diagnostic algorithm may divert patients away from a biopsy with an accurate, non-invasive diagnosis. A supplemental diagnostic algorithm could potentially reduce the number of costly biopsies and may prevent biopsy-caused morbidity and mortality. Much remains unknown about the efficacy and long-term impacts of using machine learning algorithms to diagnose IPF.

We conducted a cost-effectiveness analysis of a novel machine learning algorithm, Fibresolve™, and a genomic classifier, Envisia™, as supplements to an MDD where a high degree of confidence in a diagnosis is lacking to determine the costs and benefits of alternative diagnostics for IPF. The machine learning algorithm has been shown to improve the classification of IPF cases compared to MDD alone without the use of invasive diagnostic measures [29]. The classifier algorithm uses CT imaging and is targeted to serve as an adjunct to MDD in confirming cases of IPF non-invasively. Details of the development of the algorithm are available elsewhere [29]. This model results in a binary classification related to IPF diagnosis, and does not evaluate simply usual interstitial pneumonia (UIP) patterns. The genomic classifier has also been shown to serve as a viable alternative to surgical lung biopsy, and details are available elsewhere [23, 24]. This cost-effectiveness analysis provides clinicians and health systems with information on the potential costs and effects of novel diagnostics for IPF, a high cost chronic illnesses.

## Methods

### Model overview

**Fig. 1:**
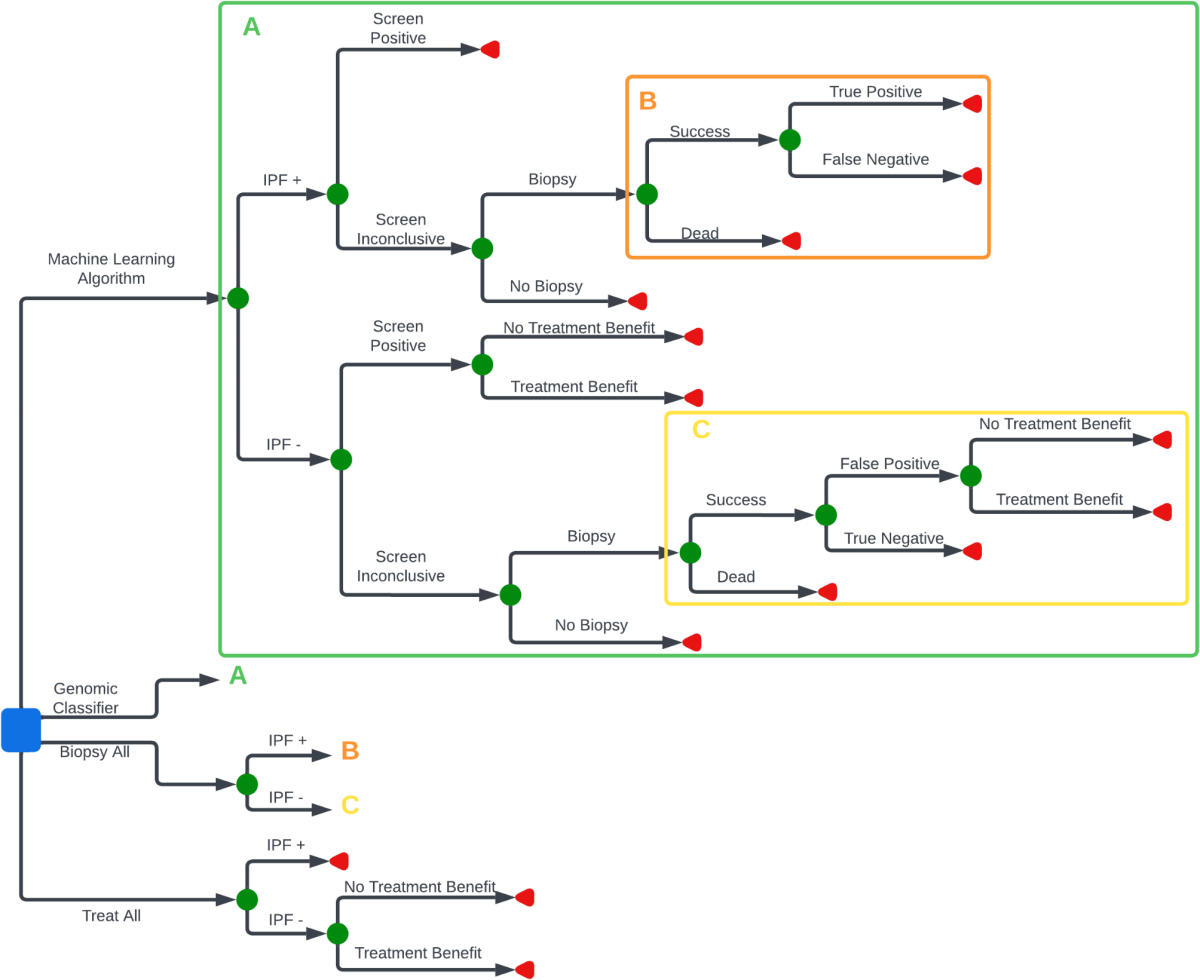
Model schematic. Legend: Blue square represents a decision node which indicates the point at which a decision is made; green circle represents a probability node at which point there are multiple uncertain outcomes; red triangle represents a terminal node at which point there is a final outcome which includes both costs and health outcomes. Green A indicates that the area of the tree not shown uses the same structure presented in the green box with parameters updated for the genomic classifier. Orange B indicates that the area of the tree not shown uses the same structure presented in the orange box with parameters upated for the biopsy-all strategy. Yellow C indicates that the area of the tree not shown uses the same structure presented in the yellow box with parameters upated for the biopsy-all strategy

We developed a decision analytic model to evaluate the effects of a machine learning algorithm and genomic classifier for the diagnosis of IPF in patients with chronic lung disease with probable to indeterminate UIP patterns based on chest computed tomography (Fig. 1). We adopted a health sector perspective and focused the analysis on patients undergoing dedicated work-up for ILD for whom an MDD had residual uncertainty about the diagnostic subtype (e.g. IPF, hypersensitivity pneumonitis, etc.), before the use of invasive procedures. The cost-effectiveness impact inventory is presented in Appendix 1 [30]. We compared the machine learning algorithm and genomic classifier to two hypothetical strategies that represent opposite ends of the theoretical spectrum with respect to IPF management – a biopsy-all strategy and a treat-all strategy – providing the full range of potential costs and benefits. The scenarios reflect the use of the machine learning algorithm and genomic classifier as a supplement to MDD, where the algorithm and classifier provide additional information on cases the MDD is unable to classify.

The model is simulated using Tree Age Pro Health Care 2022. Individuals in the algorithm and genomic classifier arms who were classified as having IPF were allocated to true positive or false positive states. Those with inconclusive diagnoses from these first-line diagnostics may receive a biopsy. If patients in this group did not undergo a biopsy they were not treated and received costs and benefits associated with a true negative or false negative state. Individuals who underwent a biopsy, in either the algorithm, genomic classifier or biopsy arms, faced a risk of death from the biopsy. Patients who underwent biopsy and did not die were allocated to either a true positive, false positive, true negative, or false negative state and accrued treatment benefits, harms, and costs accordingly. True positives received treatment for IPF using nintedanib, a standard-of-care antifibrotic therapy. Negative cases were provided symptom management, assuming that both true and false negatives had a similar prognosis. In the treat-all arm, all patients were in either a true positive or false positive state and accrued respective treatment costs, benefits, and harms. We assumed that all states were final and that no additional follow-up care would later identify false negative or false positive cases.

### Model inputs

Model input parameters are presented in Table 1.

**Table 1:** Model inputs

Input Description	Base Value	Uncertainty Rage^a^	PSA Distribution	PSA Parameters^b^	Source
*Costs*					
Algorithm	$5,000	[500–10000]	Log Normal	Mean: $5,000 Median: $4,900	Imvaria, Inc.
Genomic Classifier	$5,793	[500–10000]	Log Normal	Mean $5,793 Median: $5,500	Veracyte, Inc. [31]
Bronchoscopy	$11,170	$4,181 (Outpatient IQR) $33,838 (Inpatient IQR) [872 − 35,039]	Log Normal	Mean $11,170 Median: $5,689	Estimated from Chui et al. [20] based on weighted average of inpatient and outpatient
Surgical lung biopsy	$42,373	$30,650 (IQR) [27,048−57,698]	Log Normal	Mean: $42,373 Median: $34,482	Mean estimated from Chui et al. [20]
Symptom management	$84,073	[10,000–120,000]	Log Normal	Mean: $84,073 Median: $72,000	Dempsy et al. [15]
Lifetime treatment costs with Nintedanib	$711,579	[100,000–900,000]	Log Normal	Mean: $711,579 Median: $575,000	Dempsy et al. [15]
*Probabilities*					
Inconclusive algorithm result is referred to biopsy	0.75	[0–1]	Beta	Mean: 0.75 SD: 0.15	Assumption
Sensitivity of the algorithm	0.53	0.41–0.6 (CI) [0.41–0.65]	Beta	Mean: 0.53 SD: 0.059	Maddali et al. [29]
Specificity of the algorithm	0.86	0.77–0.93 (CI) [0.77–0.93]	Beta	Mean: 0.86 SD: 0.040	Maddali et al. [29]
Sensitivity of the genomic classifier	0.68	0.55–0.73 (CI) [0.55–0.73]	Beta	Mean: 0.68 SD: 0.045	Kheir, et al. [32]
Specificity of the genomic classifier	0.92	0.81–0.95 (CI) [0.81–0.95]	Beta	Mean: 0.92 SD: 0.035	Kheir, et al. [32]
Biopsy results in death	0.064	0.061–0.067 (CI) [0.01–0.17]	Beta	Mean: 0.064 SD: 0.02	Hutchinson et al. [21]
Biopsy sensitivity	0.75	[0.5–1]	Beta	α: 27 β: 7	Shih et al. [33]
Biopsy specificity	0.90	[0.8–1]	Beta	Mean: 0.9 SD: 0.05	Assumption
False positive case gets treatment benefit, algorithm or biopsy arm	0	[0–0.4]	Not included in PSA	Not included in PSA	Assumption
False positive case gets treatment benefit, treat-all arm	0	[0–0.2]	Not included in PSA	Not included in PSA	Assumption
Prevalence of IPF	0.404	[0.1–0.6]	Beta	α: 626 β: 922	Maddali et al. [29]
*Quality of Life*					
Decrement from undergoing surgical lung biopsy	0.013	[0–0.026]	Not included in PSA	Not included in PSA	Assumption based on Bendixen et al. [34] and Feller-Kopman et al. [35]
Death	0	-	-	-	-
QALYs gained for false negative group	3.78	[2.78–4.78]	Log Normal	Mean: 3.78 Median 3.7	Dempsy et al. [15]
QALYs gained for false positive group	3.7	[2.7–4.7]	Log Normal	Mean: 3.7 Median: 3.6	Dempsy et al. [15]
QALYs gained for true negative group	3.78	[2.85–4.85]	Log Normal	Mean: 3.78 Median 3.7	Dempsy et al. [15]
QALYs gained for true positive group	4.15	[3.15–5.15]	Log Normal	Mean: 4.15 Median: 4.05	Dempsy et al. [15]
Decrement for treatment complications	0.03	0.01 (SD) [0–0.1]	Normal	Mean: 0.03 SD: 0.01	Lloyd et al. [36]

*CI* confidence interval, *IQR* interquartile range, *SD* standard deviation

^a^Numbers in square brackets [] are the full range used for sensitivity analysis. Numbers not in brackets are those reported by the source

^b^*SD* standard deviation

#### Probabilities

We set the incidence of IPF at 40.4%, as this was the baseline incidence of IPF in the pivotal clinical study of the machine learning algorithm [29]. We varied this in sensitivity analysis.

The sensitivity and specificity of the machine learning screening algorithm came from a retrospective analysis of patients with ILD collected from US clinical sites [29]. The sensitivity and specificity of the genomic classifier came from a recent meta-analysis [32]. The sensitivity and specificity of biopsy for the identification of IPF are based on retrospective studies and assumptions consistent with previous modelling studies [33, 37]. 

Given the uncertainty in how the algorithm and classifier’s inconclusive results would be interpreted by clinicians, the probability of biopsy following an inconclusive screen by the algorithm or the genomic classifier was set initially at 0.75. We assumed the rate did not vary between the algorithm and genomic classifier arms.

The probability of dying from a surgical biopsy can range considerably from 3–16.7% [38, 39]. As a base case estimate, we used 6.7% [21].

#### Costs

Costs were set at $5,000 for the algorithm (gross price parity with genomic classifiers in similar use cases) and $16,963 for the genomic classifier (estimated based on Medicare coverage [31] and the cost of bronchoscopy [20]) in base case analyses. The total lifetime expected costs of treatment for IPF treatment with nintedanib (including all follow-up costs such as pulmonary office visits and home oxygen) and the total lifetime costs of symptom management were based on a study of the cost-effectiveness of IPF treatments in the US [15]. Additional costs included the cost of a surgical lung biopsy [20]. All costs were inflated to 2022 dollars using the Bureau of Labor Statistics Consumer Price Index for Medical Care Services.

#### Quality of life

Limited quality-of-life data exists for IPF. Rather than directly model quality of life linked to the decay of forced vital capacity, as done in CEAs of IPF treatment [15, 40–42], we used lifetime benefits from IPF treatment or symptom management as determined by a previous cost-effectiveness analysis of nintedanib and pirfenidone in the US [15]. The quality of life with treatment was used for individuals who were correctly allocated to the true positive state and false positive with treatment benefits. The quality of life with symptom management was assigned to patients allocated to negative states. The quality of life with symptom management is also used for individuals who are false positive but do not have any treatment benefit with an additional negative quality of life adjustments for potential treatment complications. The most common adverse events from nintedanib were gastrointestinal, and serious adverse events were experienced by roughly 30% of trial participants [9, 13]. To account for this, we assume 30% of patients receive a 0.1 disutility [36]. Finally, individuals who undergo a biopsy have a small quality of life decrement of 0.013 due to the biopsy procedure [34, 35]. 

### Analysis

Given the uncertainty in numerous model input parameter including how the results of the algorithm and classifier may be used by clinicians, changing costs for IPF treatments, poor data on quality of life, and impact of treatment on survival, we present average results from 10,000 probablistic simulations in lieu of expected value from the model and 95% confidence intervals (95% CIs) for costs and QALYs. Distributions for model input parameters used in the Monte Carlo simulation are specified in Table 1. We compared costs and health improvements from each intervention arm. We consider the impact of interventions on total costs as well as diagnostic-only costs. Incremental cost-effectiveness ratios (ICERs) were calculated and cost-effectiveness acceptability curves, which present the probability that an intervention is deemed cost-effective at a specific threshold, were plotted. Strategies that had greater costs and fewer QALYs were dominated.

#### Scenario and sensitivity analyses

We conducted a series of scenario analyses to explore the impact that changes in model assumptions had on outcomes. Our scenario analyses (1) examine changes in base case results with 25% and 50% reductions in lifetime treatment costs of IPF given that cheaper generic treatment options are becoming available; (2) determine the potential effects of receiving treatment following an inconclusive result from the algorithm or genomic classifier without being biopsied; and (3) potential for patients in the false positive state to receive treatment benefits. Recommendations on the use of antifibrotic treatments is a rapidly evolving area, and evidence on the potential for clinical benefit from the treatment of other progressive types of ILD is limited [43]. 

To further explore the uncertainty around different model input parameters, we conducted multiple one-way sensitivity analyses where a single parameter is varied while all others are held constant. We further evaluated the impact of changes in the costs of IPF treatments, as well as examining the effect of the sensitiviy and specificity of the supplemental screening tools, the prevalence of IPF in the sample undergoing diagnostics, and the proportion of inconclusive algorithim or genomic classifier results that receive subsequent biopsy.

In addition to ICERs, sensitivity analyses are reported in terms of net monetary benefit (NMB), which provides a single numeric value of the economic benefit of an intervention, measured in dollars, where an intervention with the highest NMB is seen as most favourable. NMB is calculated by converting the health benefit of an intervention into dollars based on a willingness-to-pay per QALY gained and then subtracting the total costs of an intervention [44]. NMB provides a simple way to compare strategies directly across disparate sensitivity analyses. A negative NMB indicates that an intervention’s opportunity costs outweigh benefits, while a positive NMB indicates the intervention provides health benefits beyond its opportunity costs [44]. Given the lack of an established willingness-to-pay threshold in the United States [45], we evaluate sensitivity analyses at multiple thresholds ($50,000, $100,000, $150,000, $200,000, and $250,000 per QALY).

## Results

### Effectiveness

Our base case probabilistic analysis found biopsy-all resulted in the lowest number of QALYs (3.63, 95% CI 3.62–3.64), followed by the machine learning algorithm with 3.75 (95% CI 3.74–3.76) QALYs, the genomic classifier with 3.77 (95% CI 3.76–3.78) QALYs, and treat-all with 3.87 (95% CI 3.85–3.88) QALYs (Table 2).

**Table 2:** Cost-effectiveness of idiopathic pulmonary fibrosis diagnosis strategies with 95% confidence intervals from probabilistic analyses

Strategy	Cost (95% CI)	Incremental Cost	QALYs (95% CI)	Incremental QALYs	ICER ($/QALY)
**Biopsy-all**	$345,680 ($341,807-$349,554)		3.63 (3.62–3.64)		
**Machine Learning Algorithm**	$385,585 ($380,881-$390,290)	$39,905	3.75 (3.74–3.76)	0.12	331,069
**Genomic Classifier**	$393,020 ($388,379-$397,660)	$7,434	3.77 (3.76–3.78)	0.02	390,043
**Treat-all**	$715,949 ($705,517-$726,381)	$322,929	3.87 (3.85–3.88)	0.10	3,245,403

 The use of the machine learning algorithm and genomic classifiers were associated with small increases in true positive (1.95 and 4.62% points, respectively) and increased false positives (6.36 and 3.04% points, respectively) compared to the biopsy-all strategy (Appendix 2). The algorithm and classifier reduced biopsy-related mortality by 3.03 and 3.15% points, respectively (Appendix 3). Treat-all increased the number of true positives and led to a ten-fold increase in false positive cases compared to biopsy-all.

### Costs

Regarding diagnostic costs, biopsy-all was the most costly strategy when considering diagnostic costs only (Table 3). When compared to the biopsy-all strategy, the machine learning algorithm and genomic classifier both reduced diagnostic-only costs by $14,876 and $3,884, respectively. The treat-all strategy has no additional diagnostic costs, and as such, reduced costs by $42,088 compared to biopsy-all.

**Table 3:** Cost breakdowns of idiopathic pulmonary fibrosis diagnosis strategies with 95% confidence intervals from probabilistic analyses

Strategy	Total Costs (95% CI)	Total Diagnostic Costs (95% CI)	Supplemental Diagnostics Costs (95% CI)	Biopsy Costs (95% CI)	Symptom Management Costs (95% CI)	Treatment Costs (95% CI)
**Biopsy-all**	$345,680 ($341,807-$349,554)	$42,088 ($41,506-$42,671)	$0 ($0-$0)	$42,088 ($41,506-$42,671)	$48,474 ($47,900-$49,048)	$255,118 ($251,323-$258,913)
**Machine Learning Algorithm**	$385,585 ($380,881-$390,290)	$27,212 ($26,883-$27,541)	$5,005 ($4,985-$5,025)	$22,207 ($21,878-$22,536)	$44,057 ($43,533-$44,582)	$314,316 ($309,649-$318,983)
**Genomic Classifier**	$393,020 ($388,379-$397,660)	$38,204 ($37,741-$38,666)	$16,807 ($16,471-$17,143)	$21,397 ($21,081-$21,713)	$44,684 ($44,154-$45,215)	$310,132 ($305,546-$314,718)
**Treat-all**	$715,949 ($705,517-$726,381)	$0 ($0-$0)	$0 ($0-$0)	$0 ($0-$0)	$0 ($0-$0)	$715,949 ($705,517-$726,381)

Conversely, when incorporating both long-term treatment and diagnostic costs and benefits, biopsy-all was the least costly strategy with average lifetime costs of $345,680 (95% CI $341,807-$349,554) per patient (Tables 2 and 3, Appendix 2). The total lifetime costs with the machine learning algorithm was the next least costly intervention, with average lifetime costs of $385,585 (95% CI $380,881-$390,290) per patient followed by the genomic classifier at $393,020 (95% CI $388,379-$397,660) per patient and treat-all with average lifetime costs of $715,949 (95% CI $705,517-$726,381) per patient. In each intervention pathway, total lifetime costs increase as more individuals are allocated to treatment states in the model.

### Cost-effectiveness of long-term treatment plus diagnostic options

ICERs for each of the intervention pathways from our probabilistic analysis with a health care sector perspective are reported in Table 2. Compared to a biopsy-all diagnostic strategy, the machine learning diagnostic algorithm pathway had an ICER of $331,069 per QALY when including the value of long-term treatment and diagnostic costs and benefits. The genomic classifier diagnostic pathway had an ICER of $390,043 per QALY compared to the algorithm pathway. Finally, the treat-all pathway had an ICER of $3,245,403 per QALY compared to the genomic classifier diagnostic pathway.

**Fig. 2:**
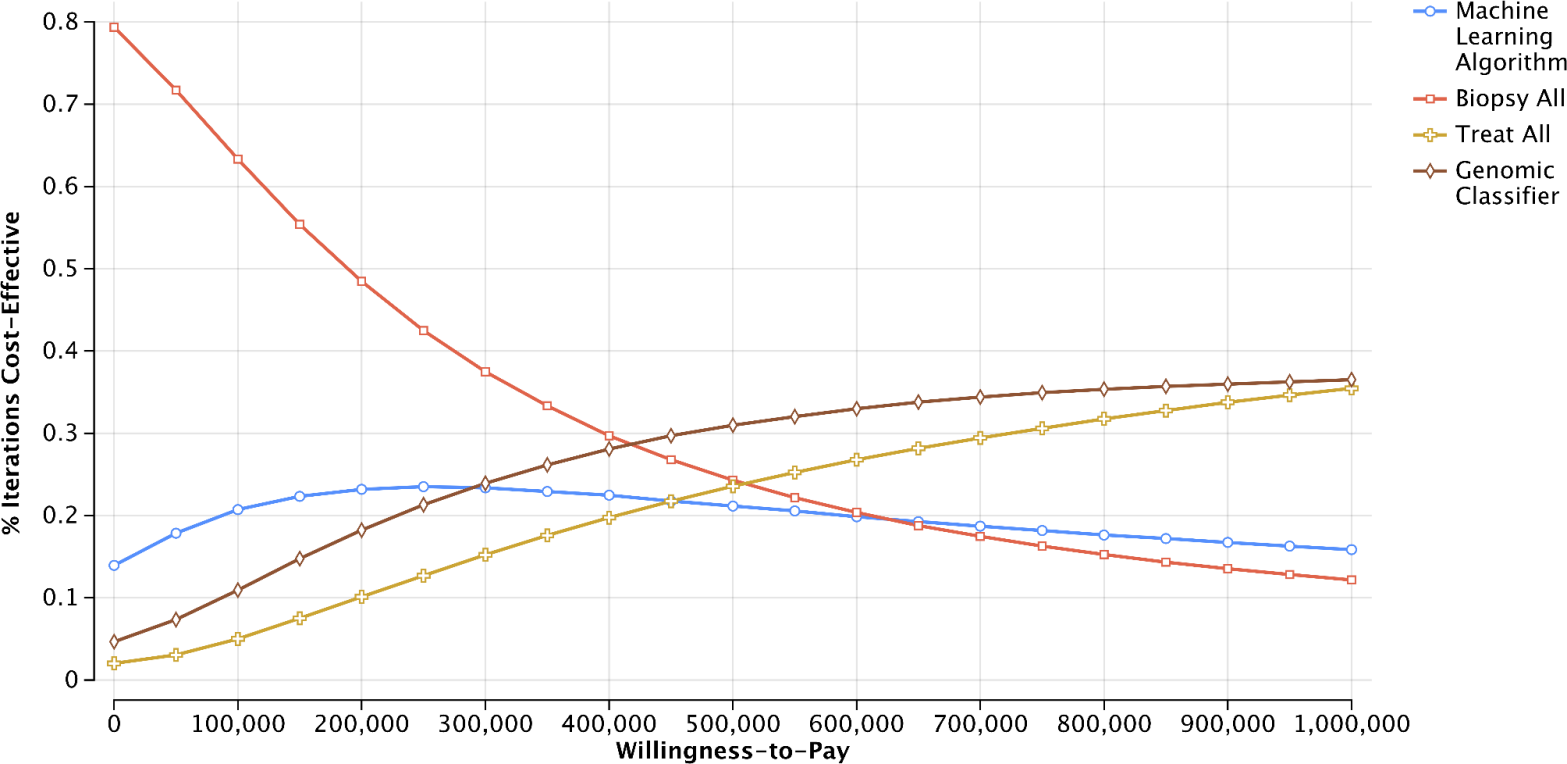
Cost-effectiveness acceptability curve. Legend: The figure presents probability that a given intervention is deemed cost-effectiveness based at a given willingness-to-pay threshold. The probability of being deemed cost-effective is based on 10,000 Monte Carlo simulations of the model

The cost-effectiveness acceptability curve illustrates the uncertainty regarding the cost-effectiveness of these intervention pathways (Fig. 2). At low willingness-to-pay levels, biopsy-all is the most attractive diagnostic and treatment selection strategy. As willingness-to-pay increases, biopsy-all becomes increasingly less attractive. Yet, even at a willingness-to-pay threshold as high as $250,000/QALY, the machine learning algorithm and genomic classifier approaches were unlikely to be deemed cost-effective with similar probabilities (23.1% and 21.6%, respectively), while the biopsy-all strategy had a 42.1% chance of being cost-effective.

### Scenario analyses

Our scenario analyses that examine the effect of reduced lifetime IPF treatment costs found increased probability of cost-effectiveness for the machine learning algorithm, genomic classifier, and treat-all strategies. With a 25% reduction in lifetime treatment costs, the low probability that the machine learning algorithm and the genomic classifier are cost-effective is relatively unchanged (Appendix 4). Reductions of treatment costs by 50% result in more favourable ICERs for the machine learning algorithm and an increased probability of cost-effectiveness for the treat-all strategy (Appendix 4). In our one-way sensitivity analysis of lifetime treatment costs for IPF, we found that with lifetime treatment costs between ~$420,000 and ~$180,000 the machine learning algorithm diagnostic pathway may be cost-effective (Appendix 5). At lifetime treatment costs below roughly $177,000, treat-all becomes the most cost-effective strategy.

Our analyses which examined potential treatment use among those with inconclusive diagnostic results and treatment benefits for individuals with false positive results did not find meaningful improvements in cost-effectiveness (results not shown).

### Sensitivity analyses

#### Inconclusive algorithm results referred to biopsy

We examined the change in net monetary benefit as the probability that an inconclusive algorithm result is referred to biopsy ranges from 0 to 1 (Appendix 6). At high willingness-to-pay thresholds, the machine learning biopsy and genomic classifier become more cost-effective than biopsy all when the biopsy referral rate is less than 0.66. With referral rates reduced further, the machine learning algorithm and genomic classifier become increasingly attractive even at lower willingness-to-pay thresholds. At lower levels, these strategies reduce biopsy costs, biopsy-related deaths, and the number of individuals who are allocated to high-cost treatment arms.

When examining diagnostic costs alone, the use of the machine learning algorithm reduced diagnostic costs compared to the biopsy-all strategy by $37,373 to $7,609, depending on the biopsy referral rate. Even when all inconclusive results were referred for biopsy, the machine learning algorithm reduced overall diagnostic costs by $7,609. Similar results were seen for the genomic classifier. When no inconclusive results were referred for biopsy, the genomic classifier pathway reduced diagnostic costs by $25,410. However, when all inconclusive results were referred for biopsy, the genomic classifier pathway increased diagnostic costs by $3,302.

#### Sensitivity and specificity of supplemental diagnostics

Given the similarities in the test characteristics of the machine learning algorithm and genomic classifier, the results of our analyses are dependent on the sensitivity and specificity of these tools. In multiple instances when ranging the sensitivity and specificity of the supplemental screening modalities across their 95% confidence intervals, the machine learning algorithm is dominated (either absolutely, where costs are higher and outcomes lower, or through extended dominance, where the next most effective intervention has a lower ICER) by the genomic classifier as sensitivity and specificity change (Appendix 7).

#### Prevalence of IPF

Finally, we examined the effect of changes in the prevalence of IPF in the sample undergoing supplemental diagnostics (Appendix 8). As the prevalence of IPF increases, the ICER for the machine learning algorithm diagnostic pathway becomes increasingly favourable. However, it is not cost-effective even with a high willingness-to-pay thresholds and 60% prevalence of IPF, the upper end of our sensitivity analysis. At the lower end (30% prevalence), the machine learning algorithm pathway is dominated by the genomic classifier pathway given the algorithm’s higher false positive rate.

## Discussion

Our analysis highlights the complicated role that diagnostic technologies play in identifying diseases with limited treatment options. IPF is challenging to diagnose and expensive to treat [14, 15, 17]. Use of the machine learning algorithm reduced diagnostic costs by $14,876 in the base case, ranging from $7,609 to $37,373 in sensitivity analyses. The genomic classifier also reduced diagnostic costs, but by a smaller margin. These results are based on the effects of the supplemental diagnostic tools in the target population and reducition in surgical lung biopies following the use of supplemental diagnostics. Both the machine learning algorithm and genomic classifier reduced biopsies and biopsy-related deaths compared to a biopsy-all strategy.

When incorporating treatment effects into a comprehensive assessment of long-term costs and benefits of novel diagnostic tools, we find that the machine learning algorithm and genomic classifier have high ICERs relative to the biopsy all strategy. High costs were driven in large part due to the high costs and limited impact of treatment, as has been documented in other cost-effectiveness analyses [15]. As a corollary, the biopsy-all strategy at the current costs for IPF treatments was overall the most cost-effective strategy given greatest selectivity in treatment. The treat-all strategy was least cost-effective.

Sensitivity analyses illustrate that the main driver of cost-effectiveness was the high cost and poor cost-effectiveness of IPF treatment. Across multiple sensitivity analyses, we found reductions in ICERs when more patients were diverted from the high-cost treatments, such as lower sensitivity of the supplemental diagnostic tools and lower rates of biopsy referral. As treatment costs lower, ICERs become more favourable and other parameters, such as quality of life for false positives and true negatives, become increasingly important. Given the novelty of machine learning diagnostics there is little evidence for how such tools might be reimbursed as such our base case reimbursement cost was based on an assumption. However, in sensitivity analysis which explore lower costs in line with other analyses of machine learning tools [46–48] we found limited impact on the ICER unless treatment costs were lower.

Given that IPF treatments are currently accepted in clinical practice and recent guidelines suggest a greater emphasis on treatment over surgical lung biopsy [49], perhaps high ICERs are not a barrier to the adoption of new interventions in this clinical space. Alternatively, a higher ICER threshold could be warranted in this setting of rare conditions and diseases with limited treatment options in order to spur innovation [50]. Previous work has found that lower-cost treatments with similar efficacy may be cost-effective [15]. Generic IPF treatments with lower costs are already available. The cost of generic Pirfenidone is considerably less than the brand name. Additionally, Medicare announced in January of 2025 that nintedanib, one of the primary treatments for IPF and the treatment included in our analysis, is part of the next round of price negotiations. Reductions in the cost of treatment would, in turn, improve the balance of costs and benefits of new IPF screening modalities.

Our overall findings show that the genomic classifier and machine learning algorithm options reduce diagnostic costs. However, they have a low likelihood of being cost-effective due to high-cost treatments, which are themselves unlikely to be cost-effective at conventional thresholds without lower costs [15]. In terms of diagnostic considerations, results of this analysis show the potential promise of alternative diagnostic strategies for IPF in several respects. Both the machine learning algorithm and genomic classifier reduced the number of biopsies and biopsy-related deaths compared to the biopsy-all strategy. Both strategies also reduced diagnostic-related costs compared to the biopsy-all strategy. For the machine learning algorithm, diagnostic costs were reduced even when all inconclusive findings were referred for biopsy. In our base case analysis, the machine learning algorithm strategy resulted in roughly the same number of true positives as the biopsy-all strategy, while the genomic classifier strategy improved the identification of true positives. When the risk of death from surgical lung biopsy is high, the application of a diagnostic algorithm can provide additional useful information with low risk.

It is also important to consider potential additional benefits that were not explicitly modelled here but that may increase the value of machine learning diagnostic algorithms. Machine learning algorithms may improve in the future. The value of scientific spillover would likely improve cost-effectiveness [51]. Future improvements in the specificity of the screening algorithm, which ensures that people are not allocated to a high-cost treatment, may result in more favourable cost-effectiveness. In addition, these algorithms may provide other benefits to patients by providing the opportunity to avoid surgery. The machine learning algorithm may also reduce delays in diagnosis for patients unable to access a medical center able to effectively diagnose IPF or have the surgical expertise for surgical lung biopsies. Delays in IPF diagnosis have been found to decrease survival [19]. Future modelling efforts should include these broader value considerations [52]. However, these may require alternative model structures which could consider the potential impact that non-invasive diagnostic tools have on reducing the time to diagnosis.

Our results are limited by numerous uncertainties and inconsistencies around how IPF is diagnosed and treated in practice. We make assumptions regarding how clinicians may continue to use biopsies following the results from the machine learning and genomic classifier diagnostic tools. Additionally, we assumed all individuals undergoing biopsy would receive a surgical lung biopsy and not transbronchial lung biopsy or cryobiopsy. While the adoption of these procedures is increasing, due to lower mortality rates, uptake is varied across the country and sensitivity is low [53, 54]. As such, our analysis used the current recommended gold standard, surgical lung biopsy [16, 17]. For treatment, we assumed that patients received treatment following the use of diagnostics and that those with negative screening results for IPF did not receive antifibrotic treatment which may not reflect clinical practice if clinicians are prescribing antifibrotics for all fibrosising interstitial lung diseases [55]. Current evidence from Medicare claims suggests that only 10.4% of IPF patients receive antifibrotics, and one quarter of those are treated prior to a coded IPF diagnosis [56]. While other studies using IPF registries have found rates of antifibrotic treatment closer to 60–70% [57, 58]. The uncertainty and inconsistency points to a need for new diagnostic approaches and additional real world evidence evaluating how IPF and ILD are being diagnosed and treated in clinical practice. Cost-effectiveness analyses should be updated once additional evidence is available.

This analysis is also limted by how we model treatment outcomes. We did not model treatment outcomes directly but instead relied on the results of a previous cost-effectiveness analysis of IPF treatment. As such, this analysis relies on how the study measured and calculated long-term quality-of-life and cost outcomes. Future analyses could try to collect cost and quality-of-life data prospectively directly from patients in a trial. However, those types of trials may not be economical for this patient population.

IPF is challenging to diagnose and treat. Novel diagnostic tools for IPF were found to reduce diagnostic costs, but are unlikely to be cost-effective in current practice given high therapeutic costs. These tools may allow for better diagnosis of IPF through reduced biopsies and diagnostic-related costs. The potential for these tools should be monitored as new IPF treatments become available and as lower-cost generic treatments emerge, which may change the cost-effectiveness result for overall disease management.

## Supplementary Information

Supplementary Material 1.

## Data Availability

All data used to develop the model are described and available in the paper. The model is available upon reasonable request from the authors.
