# Supplementary material for: Cost-effectiveness of novel diagnostic tools for idiopathic pulmonary fibrosis in the United States
Source: BMC Health Serv Res. 2025 Mar 15;25:385. doi: 10.1186/s12913-025-12506-1 (PMC11909868; doi:10.1186/s12913-025-12506-1)
Supplement: Supplementary file 1 — Supplementary Material 1. [file 12913_2025_12506_MOESM1_ESM.docx]

Appendix: Cost-Effectiveness of a Novel Machine Learning Algorithm to Diagnose Idiopathic Pulmonary Fibrosis

**Appendix 1: Cost-Effectiveness Analysis Impact Inventory**

**
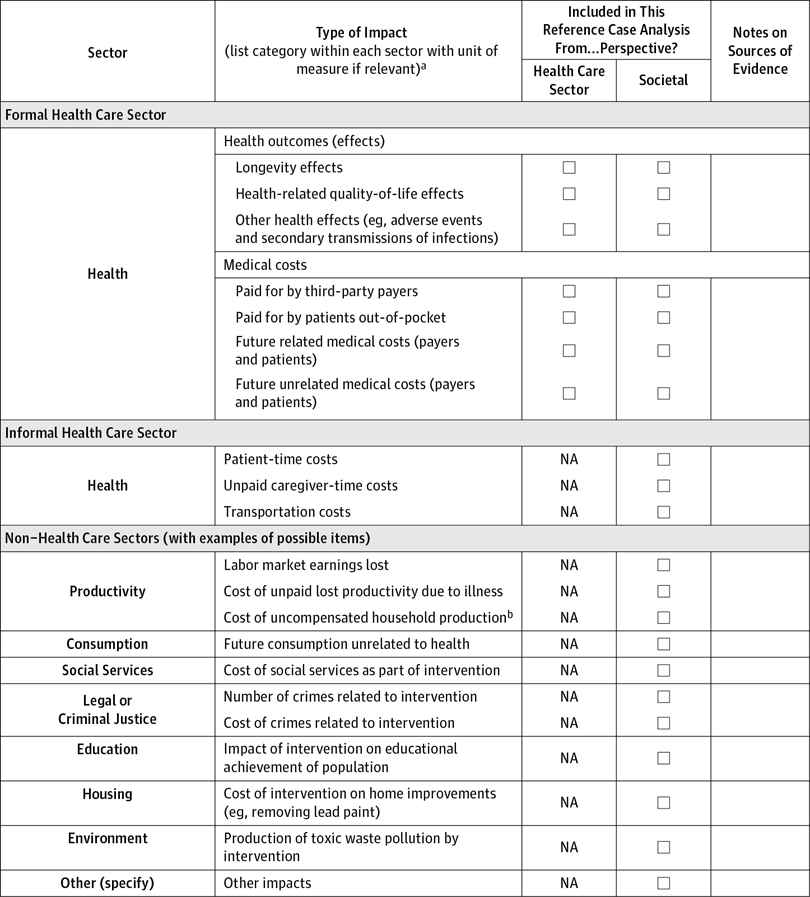
**

x

x

x

x

x

x

**Appendix 2: Final State Allocations in Base Case Analysis**

| **Strategy** | **True Positive** | **True Negative** | **False Negative** | **False Positive, No Benefit** | **False Positive, Benefit** | **Death, Biopsy** |
| --- | --- | --- | --- | --- | --- | --- |
| **Biopsy All** | 0.30 | 0.50 | 0.08 | 0.06 | 0 | 0.06 |
| **Machine Learning Algorithm** | 0.32 | 0.45 | 0.08 | 0.12 | 0 | 0.03 |
| **Genomic Classifier** | 0.35 | 0.48 | 0.05 | 0.09 | 0 | 0.03 |
| **Treat All** | 0.40 | 0.00 | 0.00 | 0.60 | 0 | 0.00 |

**Appendix 3: Biopsy Utilization Rates in Base Case Analysis**

| **Strategy** | **Biopsies** |
| --- | --- |
| **Biopsy All** | 1.00 |
| **Machine Learning Algorithm** | 0.53 |
| **Genomic Classifier** | 0.51 |
| **Treat All** | 0.00 |

**Appendix 4: Scenario Analysis with 25% and 50% Reductions to Lifetime IPF Treatment Costs**

Figure 1: Cost-Effectiveness Acceptability Curve with 25% Reduction in Lifetime IPF Treatment Costs

**
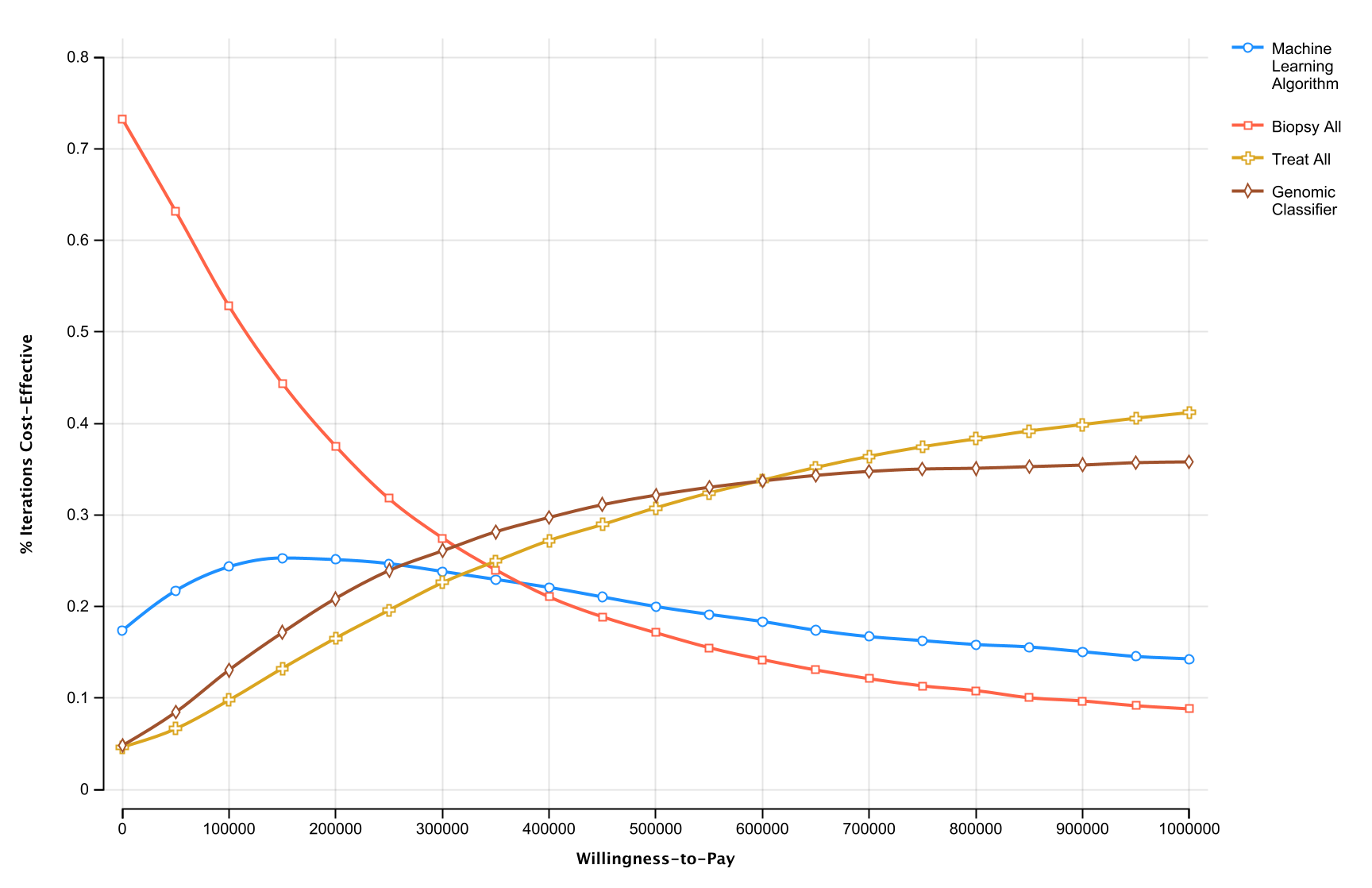
**

Table 1: Cost-Effectiveness of Idiopathic Pulmonary Fibrosis Diagnosis Strategies with a 25% Reduction in Lifetime Treatment Costs

| **Strategy** | **Cost ($)** | **Incremental Cost ($)** | **QALYs** | **Incremental QALYs** | **ICER ($/QALY)** |
| --- | --- | --- | --- | --- | --- |
| **Biopsy-all** | 282,258 | - | 3.63 | - | - |
| **Machine Learning Algorithm** | 307,658 | 25,401 | 3.75 | 0.12 | 208,884 |
| **Genomic Classifier** | 315,714 | 8,055 | 3.77 | 0.02 | 445,511 |
| **Treat-all** | 536,229 | 220,516 | 3.87 | 0.10 | 2,173,292 |

Table 2: Cost Breakdowns of Idiopathic Pulmonary Fibrosis Diagnosis Strategies with a 25% Reduction in Lifetime Treatment Costs

| **Strategy** | **Total Costs** | **Total Diagnostic Costs** | **Supplemental Diagnostics Costs** | **Biopsy Costs** | **Symptom Management Costs** | **Treatment Costs** |
| --- | --- | --- | --- | --- | --- | --- |
| **Biopsy-all** | 282,258 | 42,168 | - | 42,168 | 48,903 | 191,187 |
| **Machine Learning Algorithm** | 307,658 | 27,245 | 4,988 | 22,257 | 44,346 | 236,068 |
| **Genomic Classifier** | 315,714 | 38,292 | 16,801 | 21,491 | 45,061 | 232,361 |
| **Treat-all** | 536,229 | - | - | - | - | 536,229 |

Figure 2: Cost-Effectiveness Acceptability Curve with 50% Reduction in Lifetime IPF Treatment Costs

**
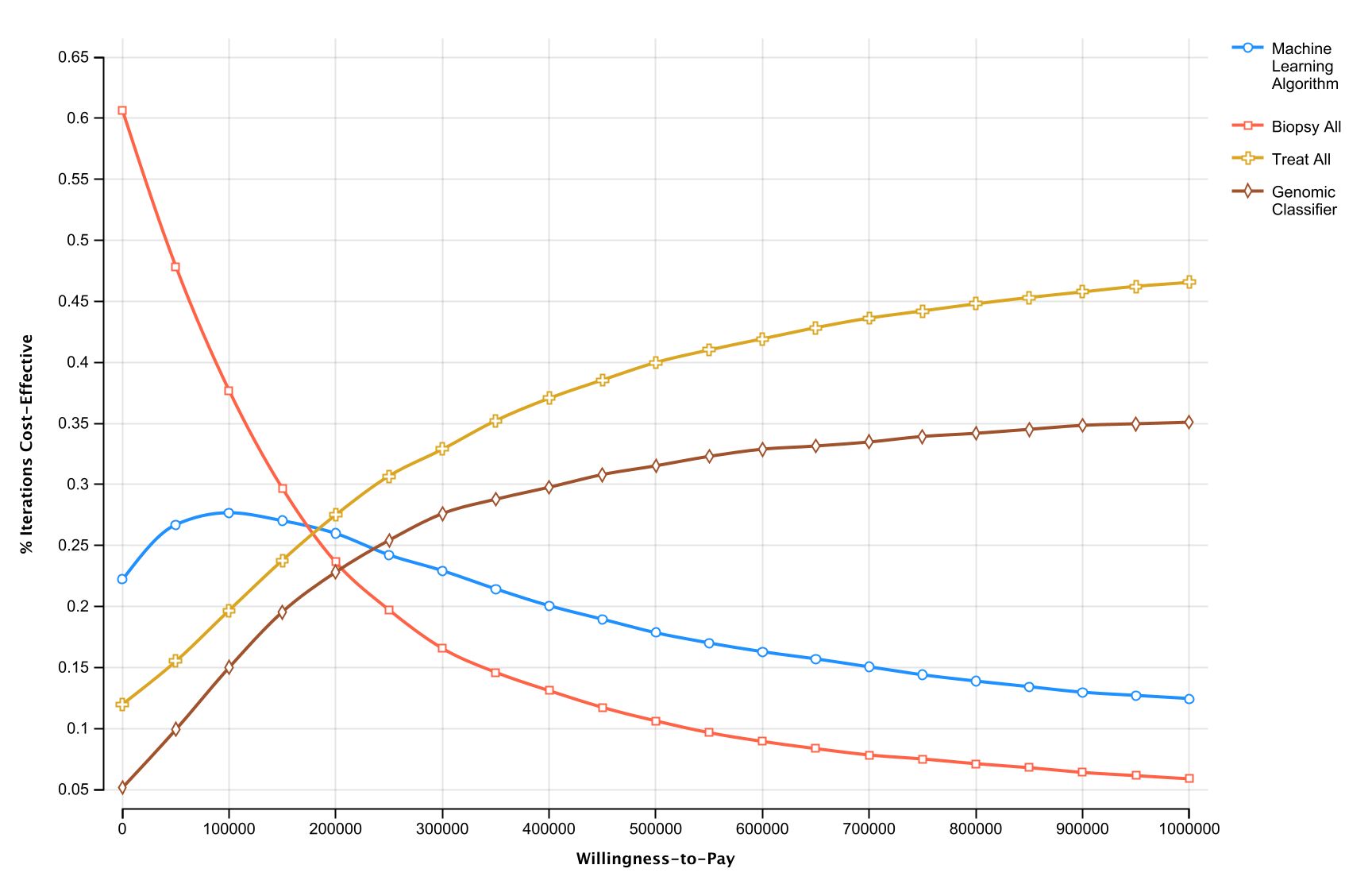
**

Table 3: Cost-Effectiveness of Idiopathic Pulmonary Fibrosis Diagnosis Strategies with a 50% Reduction in Lifetime Treatment Costs

| **Strategy** | **Cost ($)** | **Incremental Cost ($)** | **QALYs** | **Incremental QALYs** | **ICER ($/QALY)** |
| --- | --- | --- | --- | --- | --- |
| **Biopsy-all** | 217,994 | - | 3.62 | - | - |
| **Machine Learning Algorithm** | 228,317 | 10,323 | 3.74 | 0.12 | 86,307 |
| **Genomic Classifier** | 237,298 | 8,981 | 3.76 | 0.02 | 529,882 |
| **Treat-all** | 356,550 | 119,252 | 3.86 | 0.10 | 1,253,819 |

Table 4: Cost Breakdowns of Idiopathic Pulmonary Fibrosis Diagnosis Strategies with a 50% Reduction in Lifetime Treatment Costs

| **Strategy** | **Total Costs** | **Total Diagnostic Costs** | **Supplemental Diagnostics Costs** | **Biopsy Costs** | **Symptom Management Costs** | **Treatment Costs** |
| --- | --- | --- | --- | --- | --- | --- |
| **Biopsy-all** | 217,994 | 42,176 | - | 42,176 | 48,511 | 127,307 |
| **Machine Learning Algorithm** | 228,317 | 27,255 | 4,990 | 22,265 | 44,061 | 157,002 |
| **Genomic Classifier** | 237,298 | 37,872 | 16,406 | 21,466 | 44,683 | 154,743 |
| **Treat-all** | 356,550 | - | - | - | - | 356,550 |

**Appendix 5: Sensitivity Analysis on Lifetime IPF Treatment Costs**

Figure 1: Net Monetary Benefit by Lifetime Cost of IPF Treatment at Various Willingness-to-Pay Thresholds
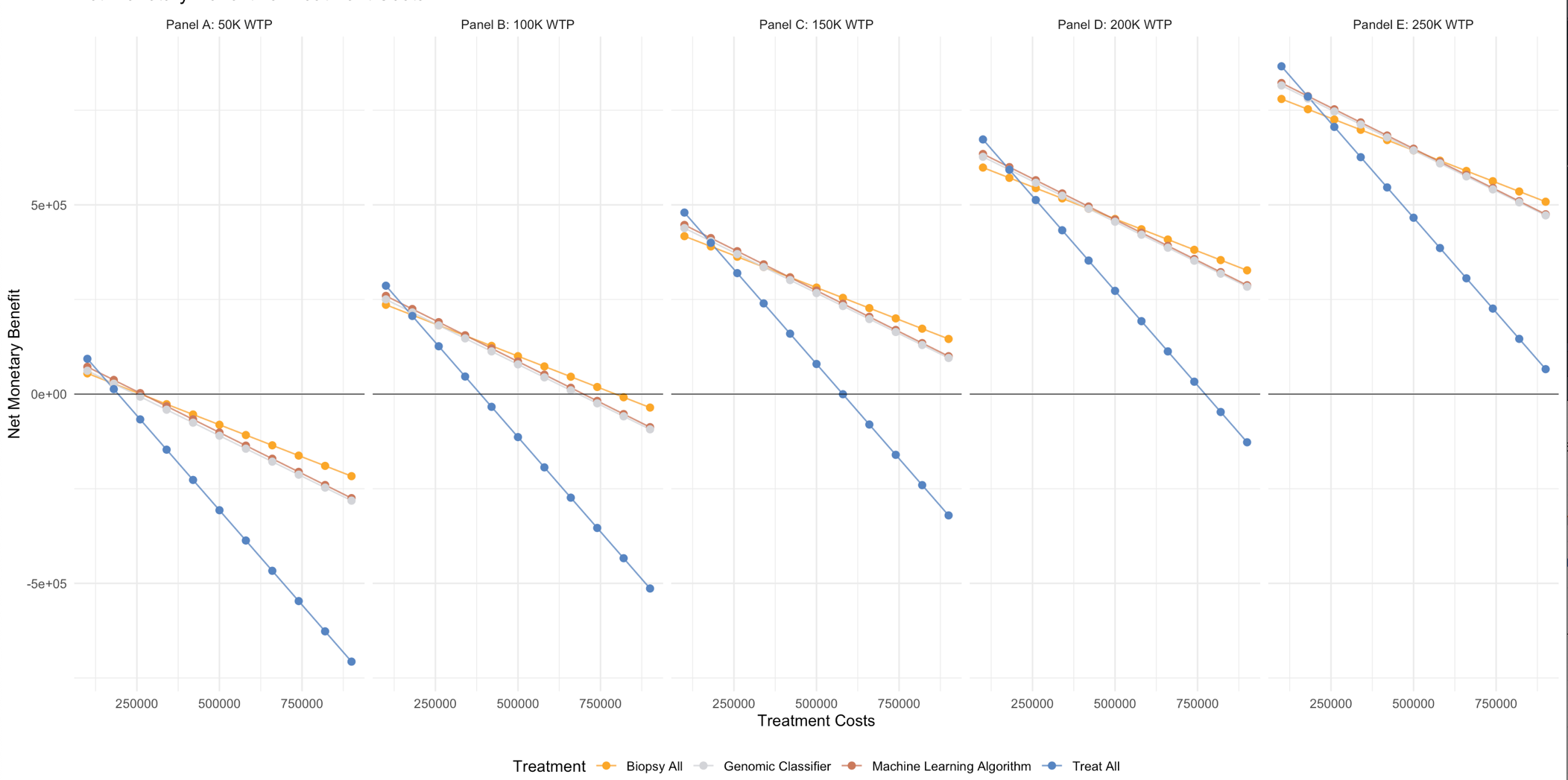


Figure Legend: The figure displays the net monetary benefit of each strategy at varying levels of lifetime IPF treatment costs using various willingness-to-pay thresholds (WTP) for the monetary value of a QALY gained.

Table 1: Incremental Cost-Effectiveness Ratio and Net Monetary Benefit by Treatment Cost

| **Treatment Cost** | **Strategy** | **Total Cost ($)** | **Incremental Cost ($)** | **Effectiveness** | **Incremental Effectiveness** | **ICER ($/QALY)** | **NMB, WTP $50K per QALY ($)** | **NMB, WTP $100K per QALY ($)** | **NMB, WTP $150K per QALY ($)** | **NMB, WTP $200K per QALY ($)** | **NMB, WTP $250K per QALY ($)** | **Dominance** |
| --- | --- | --- | --- | --- | --- | --- | --- | --- | --- | --- | --- | --- |
| 100,000 | Treat All | 100,000 | - | 3.86 | - | - | 93,196 | 286,392 | 479,588 | 672,784 | 865,980 |  |
| 100,000 | Machine Learning Algorithm | 115,466 | 15,466 | 3.75 | -0.11 | -134,837 | 71,995 | 259,456 | 446,916 | 634,377 | 821,838 | Absolute |
| 100,000 | Biopsy All | 126,471 | 26,471 | 3.62 | -0.24 | -110,660 | 54,765 | 236,000 | 417,236 | 598,471 | 779,707 | Absolute |
| 100,000 | Genomic Classifier | 126,668 | 26,668 | 3.77 | -0.1 | -278,670 | 61,743 | 250,155 | 438,566 | 626,978 | 815,389 | Absolute |
| 180,000 | Machine Learning Algorithm | 150,147 | - | 3.75 | - | - | 37,314 | 224,775 | 412,235 | 599,696 | 787,157 |  |
| 180,000 | Biopsy All | 153,622 | 3,475 | 3.62 | -0.12 | -27,910 | 27,613 | 208,849 | 390,084 | 571,320 | 752,555 | Absolute |
| 180,000 | Genomic Classifier | 160,984 | 10,837 | 3.77 | 0.02 | 570,121 | 27,427 | 215,838 | 404,250 | 592,661 | 781,072 | Extended |
| 180,000 | Treat All | 180,000 | 29,853 | 3.86 | 0.11 | 260,259 | 13,196 | 206,392 | 399,588 | 592,784 | 785,980 |  |
| 260,000 | Biopsy All | 180,774 | - | 3.62 | - | - | 462 | 181,697 | 362,933 | 544,168 | 725,404 |  |
| 260,000 | Machine Learning Algorithm | 184,828 | 4,055 | 3.75 | 0.12 | 32,565 | 2,633 | 190,094 | 377,554 | 565,015 | 752,476 |  |
| 260,000 | Genomic Classifier | 195,301 | 10,472 | 3.77 | 0.02 | 550,948 | -6,890 | 181,521 | 369,933 | 558,344 | 746,755 |  |
| 260,000 | Treat All | 260,000 | 64,699 | 3.86 | 0.1 | 676,093 | -66,804 | 126,392 | 319,588 | 512,784 | 705,980 |  |
| 340,000 | Biopsy All | 207,925 | - | 3.62 | - | - | -26,690 | 154,546 | 335,781 | 517,017 | 698,252 |  |
| 340,000 | Machine Learning Algorithm | 219,509 | 11,584 | 3.75 | 0.12 | 93,041 | -32,048 | 155,413 | 342,873 | 530,334 | 717,795 |  |
| 340,000 | Genomic Classifier | 229,617 | 10,108 | 3.77 | 0.02 | 531,775 | -41,206 | 147,205 | 335,617 | 524,028 | 712,439 |  |
| 340,000 | Treat All | 340,000 | 110,383 | 3.86 | 0.1 | 1,153,475 | -146,804 | 46,392 | 239,588 | 432,784 | 625,980 |  |
| 420,000 | Biopsy All | 235,077 | - | 3.62 | - | - | -53,841 | 127,394 | 308,630 | 489,865 | 671,101 |  |
| 420,000 | Machine Learning Algorithm | 254,191 | 19,114 | 3.75 | 0.12 | 153,517 | -66,730 | 120,731 | 308,192 | 495,653 | 683,114 |  |
| 420,000 | Genomic Classifier | 263,934 | 9,744 | 3.77 | 0.02 | 512,602 | -75,523 | 112,888 | 301,300 | 489,711 | 678,122 |  |
| 420,000 | Treat All | 420,000 | 156,066 | 3.86 | 0.1 | 1,630,857 | -226,804 | -33,608 | 159,588 | 352,784 | 545,980 |  |
| 500,000 | Biopsy All | 262,228 | - | 3.62 | - | - | -80,993 | 100,243 | 281,478 | 462,714 | 643,949 |  |
| 500,000 | Machine Learning Algorithm | 288,872 | 26,643 | 3.75 | 0.12 | 213,993 | -101,411 | 86,050 | 273,510 | 460,971 | 648,432 |  |
| 500,000 | Genomic Classifier | 298,251 | 9,379 | 3.77 | 0.02 | 493,428 | -109,840 | 78,571 | 266,983 | 455,394 | 643,805 |  |
| 500,000 | Treat All | 500,000 | 201,749 | 3.86 | 0.1 | 2,108,239 | -306,804 | -113,608 | 79,588 | 272,784 | 465,980 |  |
| 580,000 | Biopsy All | 289,380 | - | 3.62 | - | - | -108,144 | 73,091 | 254,327 | 435,562 | 616,798 |  |
| 580,000 | Machine Learning Algorithm | 323,553 | 34,173 | 3.75 | 0.12 | 274,469 | -136,092 | 51,369 | 238,829 | 426,290 | 613,751 |  |
| 580,000 | Genomic Classifier | 332,567 | 9,015 | 3.77 | 0.02 | 474,255 | -144,156 | 44,255 | 232,667 | 421,078 | 609,489 |  |
| 580,000 | Treat All | 580,000 | 247,433 | 3.86 | 0.1 | 2,585,620 | -386,804 | -193,608 | -412 | 192,784 | 385,980 |  |
| 660,000 | Biopsy All | 316,531 | - | 3.62 | - | - | -135,296 | 45,940 | 227,175 | 408,411 | 589,646 |  |
| 660,000 | Machine Learning Algorithm | 358,234 | 41,703 | 3.75 | 0.12 | 334,944 | -170,773 | 16,688 | 204,148 | 391,609 | 579,070 |  |
| 660,000 | Genomic Classifier | 366,884 | 8,650 | 3.77 | 0.02 | 455,082 | -178,473 | 9,938 | 198,350 | 386,761 | 575,172 |  |
| 660,000 | Treat All | 660,000 | 293,116 | 3.86 | 0.1 | 3,063,002 | -466,804 | -273,608 | -80,412 | 112,784 | 305,980 |  |
| 740,000 | Biopsy All | 343,683 | - | 3.62 | - | - | -162,447 | 18,788 | 200,024 | 381,259 | 562,495 |  |
| 740,000 | Machine Learning Algorithm | 392,915 | 49,232 | 3.75 | 0.12 | 395,420 | -205,454 | -17,993 | 169,467 | 356,928 | 544,389 |  |
| 740,000 | Genomic Classifier | 401,201 | 8,286 | 3.77 | 0.02 | 435,909 | -212,790 | -24,379 | 164,033 | 352,444 | 540,855 |  |
| 740,000 | Treat All | 740,000 | 338,799 | 3.86 | 0.1 | 3,540,384 | -546,804 | -353,608 | -160,412 | 32,784 | 225,980 |  |
| 820,000 | Biopsy All | 370,834 | - | 3.62 | - | - | -189,599 | -8,363 | 172,872 | 354,108 | 535,343 |  |
| 820,000 | Machine Learning Algorithm | 427,596 | 56,762 | 3.75 | 0.12 | 455,896 | -240,135 | -52,674 | 134,786 | 322,247 | 509,708 | Extended |
| 820,000 | Genomic Classifier | 435,517 | 64,683 | 3.77 | 0.14 | 450,709 | -247,106 | -58,695 | 129,717 | 318,128 | 506,539 |  |
| 820,000 | Treat All | 820,000 | 384,483 | 3.86 | 0.1 | 4,017,766 | -626,804 | -433,608 | -240,412 | -47,216 | 145,980 |  |
| 900,000 | Biopsy All | 397,986 | - | 3.62 | - | - | -216,750 | -35,515 | 145,721 | 326,956 | 508,192 |  |
| 900,000 | Machine Learning Algorithm | 462,277 | 64,291 | 3.75 | 0.12 | 516,372 | -274,816 | -87,355 | 100,105 | 287,566 | 475,027 | Extended |
| 900,000 | Genomic Classifier | 469,834 | 71,848 | 3.77 | 0.14 | 500,636 | -281,423 | -93,012 | 95,400 | 283,811 | 472,222 |  |
| 900,000 | Treat All | 900,000 | 430,166 | 3.86 | 0.1 | 4,495,147 | -706,804 | -513,608 | -320,412 | -127,216 | 65,980 |  |

Table 2: Cost Breakdown by Treatment Costs

| **Treatment Cost** | **Strategy** | **Total Diagnostic Costs** | **Biopsy Costs** | **Adjunct Diagnostic Costs** | **Symptom Management Costs** | **Treatment Costs** |
| --- | --- | --- | --- | --- | --- | --- |
| 100,000 | Treat All | 42,373 | 42,373 | - | 55,478 | 100,000 |
| 100,000 | Machine Learning Algorithm | 28,612 | 23,612 | 5,000 | 49,674 | 43,351 |
| 100,000 | Biopsy All | 40,480 | 23,517 | 16,963 | 51,178 | 33,939 |
| 100,000 | Genomic Classifier | - | - | - | - | 42,896 |
| 180,000 | Machine Learning Algorithm | 42,373 | 42,373 | - | 52,921 | 78,032 |
| 180,000 | Biopsy All | 27,993 | 22,993 | 5,000 | 47,326 | 61,091 |
| 180,000 | Genomic Classifier | 39,527 | 22,564 | 16,963 | 48,340 | 77,212 |
| 180,000 | Treat All | - | - | - | - | 180,000 |
| 260,000 | Biopsy All | 42,373 | 42,373 | - | 50,363 | 88,242 |
| 260,000 | Machine Learning Algorithm | 27,373 | 22,373 | 5,000 | 44,979 | 112,714 |
| 260,000 | Genomic Classifier | 38,573 | 21,610 | 16,963 | 45,502 | 111,529 |
| 260,000 | Treat All | - | - | - | - | 260,000 |
| 340,000 | Biopsy All | 42,373 | 42,373 | - | 47,806 | 115,394 |
| 340,000 | Machine Learning Algorithm | 26,753 | 21,753 | 5,000 | 42,632 | 147,395 |
| 340,000 | Genomic Classifier | 37,620 | 20,657 | 16,963 | 42,664 | 145,846 |
| 340,000 | Treat All | - | - | - | - | 340,000 |
| 420,000 | Biopsy All | 42,373 | 42,373 | - | 45,248 | 142,545 |
| 420,000 | Machine Learning Algorithm | 26,134 | 21,134 | 5,000 | 40,285 | 182,076 |
| 420,000 | Genomic Classifier | 36,666 | 19,703 | 16,963 | 39,826 | 180,162 |
| 420,000 | Treat All | - | - | - | - | 420,000 |
| 500,000 | Biopsy All | 42,373 | 42,373 | - | 42,691 | 169,697 |
| 500,000 | Machine Learning Algorithm | 25,514 | 20,514 | 5,000 | 37,938 | 216,757 |
| 500,000 | Genomic Classifier | 35,713 | 18,750 | 16,963 | 36,988 | 214,479 |
| 500,000 | Treat All | - | - | - | - | 500,000 |
| 580,000 | Biopsy All | 42,373 | 42,373 | - | 40,133 | 196,848 |
| 580,000 | Machine Learning Algorithm | 24,894 | 19,894 | 5,000 | 35,591 | 251,438 |
| 580,000 | Genomic Classifier | 34,760 | 17,797 | 16,963 | 34,150 | 248,796 |
| 580,000 | Treat All | - | - | - | - | 580,000 |
| 660,000 | Biopsy All | 42,373 | 42,373 | - | 55,478 | 224,000 |
| 660,000 | Machine Learning Algorithm | 28,612 | 23,612 | 5,000 | 49,674 | 286,119 |
| 660,000 | Genomic Classifier | 40,480 | 23,517 | 16,963 | 51,178 | 283,112 |
| 660,000 | Treat All | - | - | - | - | 660,000 |
| 740,000 | Biopsy All | 42,373 | 42,373 | - | 52,921 | 251,151 |
| 740,000 | Machine Learning Algorithm | 27,993 | 22,993 | 5,000 | 47,326 | 320,800 |
| 740,000 | Genomic Classifier | 39,527 | 22,564 | 16,963 | 48,340 | 317,429 |
| 740,000 | Treat All | - | - | - | - | 740,000 |
| 820,000 | Biopsy All | 42,373 | 42,373 | - | 50,363 | 278,303 |
| 820,000 | Machine Learning Algorithm | 27,373 | 22,373 | 5,000 | 44,979 | 355,481 |
| 820,000 | Genomic Classifier | 38,573 | 21,610 | 16,963 | 45,502 | 351,746 |
| 820,000 | Treat All | - | - | - | - | 820,000 |
| 900,000 | Biopsy All | 42,373 | 42,373 | - | 47,806 | 305,454 |
| 900,000 | Machine Learning Algorithm | 26,753 | 21,753 | 5,000 | 42,632 | 390,162 |
| 900,000 | Genomic Classifier | 37,620 | 20,657 | 16,963 | 42,664 | 386,062 |
| 900,000 | Treat All | - | - | - | - | 900,000 |

**Appendix 6: Sensitivity Analysis on the Probability an Inconclusive Adjunct Diagnostic Result is Referred to Biopsy**

Figure 1: Net Monetary Benefit by Probability that an Inconclusive Adjunct Diagnostic Result is Referred for Biopsy


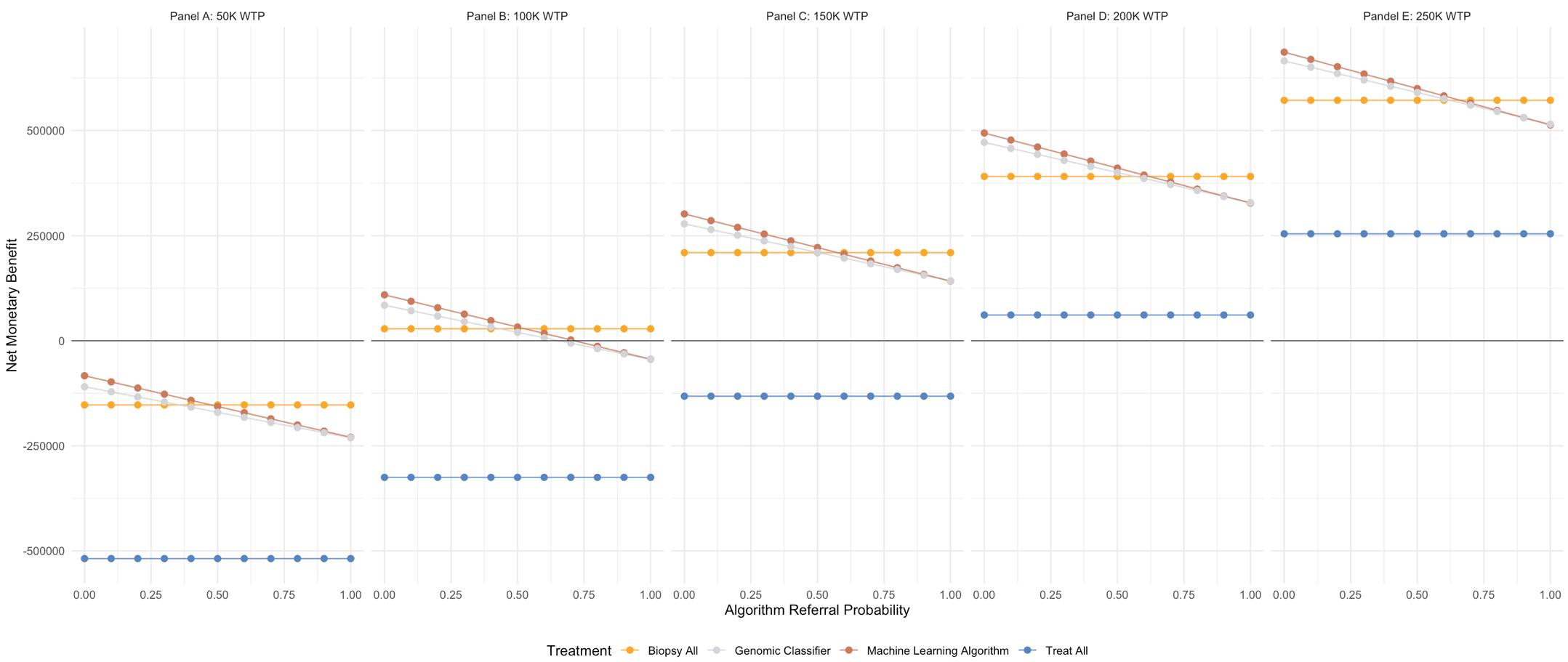


Figure Legend: The figure displays the net monetary benefit of each strategy with varying probabilities that inconclusive results from the genomic classifier and machine learning diagnostic tools are referred for surgical lung biopsy using various willingness-to-pay thresholds (WTP) for the monetary value of a QALY gained.

Table 1: Incremental Cost-Effectiveness Ratio and Net Monetary Benefit by Algorithm Referral Rate

| **Probability of Algorithm Referral** | **Strategy** | **Costs ($)** | **Incremental Cost ($)** | **Effectiveness** | **Incremental Effectiveness** | **ICER ($/QALY)** | **NMB, WTP $50K per QALY ($)** | **NMB, WTP $100K per QALY ($)** | **NMB, WTP $150K per QALY ($)** | **NMB, WTP $200K per QALY ($)** | **NMB, WTP $250K per QALY ($)** | **Dominance** |
| --- | --- | --- | --- | --- | --- | --- | --- | --- | --- | --- | --- | --- |
| 0 | Machine Learning Algorithm | 275,794 | - | 3.85 | - | - | -83,292 | 109,211 | 301,713 | 494,216 | 686,718 |  |
| 0 | Genomic Classifier | 303,344 | 27,550 | 3.88 | 0.03 | 1,045,328 | -109,524 | 84,296 | 278,116 | 471,936 | 665,756 |  |
| 0 | Biopsy All | 334,037 | 30,693 | 3.62 | -0.25 | -121,947 | -152,801 | 28,434 | 209,670 | 390,905 | 572,141 | Absolute |
| 0 | Treat All | 711,579 | 408,235 | 3.86 | -0.01 | -32,706,950 | -518,383 | -325,187 | -131,991 | 61,205 | 254,401 | Absolute |
| 0.1 | Machine Learning Algorithm | 289,767 | - | 3.84 | - | - | -97,937 | 93,893 | 285,723 | 477,553 | 669,383 |  |
| 0.1 | Genomic Classifier | 314,766 | 24,999 | 3.86 | 0.03 | 985,143 | -121,667 | 71,432 | 264,531 | 457,630 | 650,729 |  |
| 0.1 | Biopsy All | 334,037 | 19,271 | 3.62 | -0.24 | -81,220 | -152,801 | 28,434 | 209,670 | 390,905 | 572,141 | Absolute |
| 0.1 | Treat All | 711,579 | 396,813 | 3.86 | 0 | 204,328,524 | -518,383 | -325,187 | -131,991 | 61,205 | 254,401 |  |
| 0.2 | Machine Learning Algorithm | 303,740 | - | 3.82 | - | - | -112,582 | 78,576 | 269,733 | 460,891 | 652,049 |  |
| 0.2 | Genomic Classifier | 326,188 | 22,448 | 3.85 | 0.02 | 920,125 | -133,810 | 58,568 | 250,945 | 443,323 | 635,701 |  |
| 0.2 | Biopsy All | 334,037 | 7,849 | 3.62 | -0.22 | -35,221 | -152,801 | 28,434 | 209,670 | 390,905 | 572,141 | Absolute |
| 0.2 | Treat All | 711,579 | 385,391 | 3.86 | 0.02 | 23,548,747 | -518,383 | -325,187 | -131,991 | 61,205 | 254,401 |  |
| 0.3 | Machine Learning Algorithm | 317,714 | - | 3.81 | - | - | -127,228 | 63,258 | 253,743 | 444,229 | 634,715 |  |
| 0.3 | Biopsy All | 334,037 | 16,323 | 3.62 | -0.19 | -88,231 | -152,801 | 28,434 | 209,670 | 390,905 | 572,141 | Absolute |
| 0.3 | Genomic Classifier | 337,610 | 19,896 | 3.83 | 0.02 | 849,666 | -145,953 | 45,703 | 237,360 | 429,016 | 620,673 |  |
| 0.3 | Treat All | 711,579 | 373,969 | 3.86 | 0.03 | 12,146,067 | -518,383 | -325,187 | -131,991 | 61,205 | 254,401 |  |
| 0.4 | Machine Learning Algorithm | 331,687 | - | 3.8 | - | - | -141,874 | 47,940 | 237,753 | 427,567 | 617,380 |  |
| 0.4 | Biopsy All | 334,037 | 2,350 | 3.62 | -0.17 | -13,696 | -152,801 | 28,434 | 209,670 | 390,905 | 572,141 | Absolute |
| 0.4 | Genomic Classifier | 349,032 | 17,345 | 3.82 | 0.02 | 773,054 | -158,097 | 32,839 | 223,774 | 414,710 | 605,645 |  |
| 0.4 | Treat All | 711,579 | 362,547 | 3.86 | 0.05 | 8,018,654 | -518,383 | -325,187 | -131,991 | 61,205 | 254,401 |  |
| 0.5 | Biopsy All | 334,037 | - | 3.62 | - | - | -152,801 | 28,434 | 209,670 | 390,905 | 572,141 |  |
| 0.5 | Machine Learning Algorithm | 345,661 | 11,624 | 3.78 | 0.16 | 73,514 | -156,520 | 32,622 | 221,763 | 410,905 | 600,046 |  |
| 0.5 | Genomic Classifier | 360,454 | 14,794 | 3.8 | 0.02 | 689,446 | -170,240 | 19,974 | 210,189 | 400,403 | 590,617 |  |
| 0.5 | Treat All | 711,579 | 351,125 | 3.86 | 0.06 | 5,887,744 | -518,383 | -325,187 | -131,991 | 61,205 | 254,401 |  |
| 0.6 | Biopsy All | 334,037 | - | 3.62 | - | - | -152,801 | 28,434 | 209,670 | 390,905 | 572,141 |  |
| 0.6 | Machine Learning Algorithm | 359,634 | 25,597 | 3.77 | 0.14 | 176,932 | -171,165 | 17,304 | 205,774 | 394,243 | 582,712 |  |
| 0.6 | Genomic Classifier | 371,876 | 12,242 | 3.79 | 0.02 | 597,838 | -182,383 | 7,110 | 196,603 | 386,096 | 575,589 |  |
| 0.6 | Treat All | 711,579 | 339,703 | 3.86 | 0.07 | 4,586,847 | -518,383 | -325,187 | -131,991 | 61,205 | 254,401 |  |
| 0.7 | Biopsy All | 334,037 | - | 3.62 | - | - | -152,801 | 28,434 | 209,670 | 390,905 | 572,141 |  |
| 0.7 | Machine Learning Algorithm | 373,607 | 39,570 | 3.76 | 0.13 | 301,540 | -185,810 | 1,987 | 189,783 | 377,580 | 565,377 |  |
| 0.7 | Genomic Classifier | 383,298 | 9,691 | 3.78 | 0.02 | 497,024 | -194,526 | -5,754 | 183,017 | 371,789 | 560,561 |  |
| 0.7 | Treat All | 711,579 | 328,281 | 3.86 | 0.09 | 3,710,065 | -518,383 | -325,187 | -131,991 | 61,205 | 254,401 |  |
| 0.8 | Biopsy All | 334,037 | - | 3.62 | - | - | -152,801 | 28,434 | 209,670 | 390,905 | 572,141 |  |
| 0.8 | Machine Learning Algorithm | 387,581 | 53,544 | 3.74 | 0.12 | 454,594 | -200,456 | -13,331 | 173,793 | 360,918 | 548,043 | Extended |
| 0.8 | Genomic Classifier | 394,720 | 60,683 | 3.76 | 0.14 | 445,213 | -206,669 | -18,619 | 169,432 | 357,482 | 545,533 |  |
| 0.8 | Treat All | 711,579 | 316,859 | 3.86 | 0.1 | 3,079,065 | -518,383 | -325,187 | -131,991 | 61,205 | 254,401 |  |
| 0.9 | Biopsy All | 334,037 | - | 3.62 | - | - | -152,801 | 28,434 | 209,670 | 390,905 | 572,141 |  |
| 0.9 | Machine Learning Algorithm | 401,554 | 67,517 | 3.73 | 0.1 | 647,089 | -215,101 | -28,649 | 157,804 | 344,256 | 530,709 | Extended |
| 0.9 | Genomic Classifier | 406,142 | 72,105 | 3.75 | 0.12 | 591,618 | -218,813 | -31,483 | 155,846 | 343,176 | 530,505 |  |
| 0.9 | Treat All | 711,579 | 305,437 | 3.86 | 0.12 | 2,603,203 | -518,383 | -325,187 | -131,991 | 61,205 | 254,401 |  |
| 1 | Biopsy All | 334,037 | - | 3.62 | - | - | -152,801 | 28,434 | 209,670 | 390,905 | 572,141 |  |
| 1 | Machine Learning Algorithm | 415,527 | 81,491 | 3.72 | 0.09 | 896,526 | -229,747 | -43,967 | 141,814 | 327,594 | 513,374 | Extended |
| 1 | Genomic Classifier | 417,564 | 83,527 | 3.73 | 0.11 | 777,327 | -230,956 | -44,348 | 142,261 | 328,869 | 515,477 |  |
| 1 | Treat All | 711,579 | 294,015 | 3.86 | 0.13 | 2,231,530 | -518,383 | -325,187 | -131,991 | 61,205 | 254,401 |  |

Table 2: Cost Breakdown by Algorithm Referral Rate

| **Probability of Algorithm Referral** | **Strategy** | **Total Diagnostic Costs ($)** | **Biopsy Costs ($)** | **Supplemental Diagnostic Costs ($)** | **Symptom Management Costs ($)** | **Treatment Costs ($)** |
| --- | --- | --- | --- | --- | --- | --- |
| 0 | Machine Learning Algorithm | 5,000 | - | 5,000 | 59,056 | 211,737 |
| 0 | Genomic Classifier | 16,963 | - | 16,963 | 56,968 | 229,413 |
| 0 | Biopsy All | 42,373 | 42,373 | - | 50,158 | 241,505 |
| 0 | Treat All | - | - | - | - | 711,579 |
| 0.1 | Machine Learning Algorithm | 7,976 | 2,976 | 5,000 | 57,154 | 224,636 |
| 0.1 | Genomic Classifier | 19,834 | 2,871 | 16,963 | 55,409 | 239,523 |
| 0.1 | Biopsy All | 42,373 | 42,373 | - | 50,158 | 241,505 |
| 0.1 | Treat All | - | - | - | - | 711,579 |
| 0.2 | Machine Learning Algorithm | 10,953 | 5,953 | 5,000 | 55,252 | 237,535 |
| 0.2 | Genomic Classifier | 22,705 | 5,742 | 16,963 | 53,850 | 249,633 |
| 0.2 | Biopsy All | 42,373 | 42,373 | - | 50,158 | 241,505 |
| 0.2 | Treat All | - | - | - | - | 711,579 |
| 0.3 | Machine Learning Algorithm | 13,929 | 8,929 | 5,000 | 53,350 | 250,434 |
| 0.3 | Biopsy All | 42,373 | 42,373 | - | 50,158 | 241,505 |
| 0.3 | Genomic Classifier | 25,577 | 8,614 | 16,963 | 52,291 | 259,743 |
| 0.3 | Treat All | - | - | - | - | 711,579 |
| 0.4 | Machine Learning Algorithm | 16,906 | 11,906 | 5,000 | 51,448 | 263,333 |
| 0.4 | Biopsy All | 42,373 | 42,373 | - | 50,158 | 241,505 |
| 0.4 | Genomic Classifier | 28,448 | 11,485 | 16,963 | 50,732 | 269,853 |
| 0.4 | Treat All | - | - | - | - | 711,579 |
| 0.5 | Biopsy All | 42,373 | 42,373 | - | 50,158 | 241,505 |
| 0.5 | Machine Learning Algorithm | 19,882 | 14,882 | 5,000 | 49,546 | 276,232 |
| 0.5 | Genomic Classifier | 31,319 | 14,356 | 16,963 | 49,172 | 279,963 |
| 0.5 | Treat All | - | - | - | - | 711,579 |
| 0.6 | Biopsy All | 42,373 | 42,373 | - | 50,158 | 241,505 |
| 0.6 | Machine Learning Algorithm | 22,859 | 17,859 | 5,000 | 47,644 | 289,131 |
| 0.6 | Genomic Classifier | 34,190 | 17,227 | 16,963 | 47,613 | 290,073 |
| 0.6 | Treat All | - | - | - | - | 711,579 |
| 0.7 | Biopsy All | 42,373 | 42,373 | - | 50,158 | 241,505 |
| 0.7 | Machine Learning Algorithm | 25,835 | 20,835 | 5,000 | 45,742 | 302,030 |
| 0.7 | Genomic Classifier | 37,061 | 20,098 | 16,963 | 46,054 | 300,183 |
| 0.7 | Treat All | - | - | - | - | 711,579 |
| 0.8 | Biopsy All | 42,373 | 42,373 | - | 50,158 | 241,505 |
| 0.8 | Machine Learning Algorithm | 28,812 | 23,812 | 5,000 | 43,841 | 314,929 |
| 0.8 | Genomic Classifier | 39,933 | 22,970 | 16,963 | 44,495 | 310,292 |
| 0.8 | Treat All | - | - | - | - | 711,579 |
| 0.9 | Biopsy All | 42,373 | 42,373 | - | 50,158 | 241,505 |
| 0.9 | Machine Learning Algorithm | 31,788 | 26,788 | 5,000 | 41,939 | 327,827 |
| 0.9 | Genomic Classifier | 42,804 | 25,841 | 16,963 | 42,936 | 320,402 |
| 0.9 | Treat All | - | - | - | - | 711,579 |
| 1 | Biopsy All | 42,373 | 42,373 | - | 50,158 | 241,505 |
| 1 | Machine Learning Algorithm | 34,764 | 29,764 | 5,000 | 40,037 | 340,726 |
| 1 | Genomic Classifier | 45,675 | 28,712 | 16,963 | 41,377 | 330,512 |
| 1 | Treat All | - | - | - | - | 711,579 |

Table 3: Final State Allocations by Algorithm Referral Rate

| **Probability of Algorithm Referral** | **Strategy** | **True Positive** | **True Negative** | **False Positive, No Benefit** | **False Positive, Benefit** | **False Negative** | **Death, Biopsy** |
| --- | --- | --- | --- | --- | --- | --- | --- |
| 0 | Machine Learning Algorithm | 0.214 | 0.513 | 0.083 | - | 0.190 | 0.190 |
| 0 | Genomic Classifier | 0.275 | 0.548 | 0.048 | - | 0.129 | 0.129 |
| 0 | Biopsy All | 0.284 | 0.502 | 0.056 | - | 0.095 | 0.095 |
| 0 | Treat All | 0.404 | - | 0.596 | - | - | - |
| 0.1 | Machine Learning Algorithm | 0.227 | 0.504 | 0.088 | - | 0.175 | 0.175 |
| 0.1 | Genomic Classifier | 0.284 | 0.540 | 0.053 | - | 0.119 | 0.119 |
| 0.1 | Biopsy All | 0.284 | 0.502 | 0.056 | - | 0.095 | 0.095 |
| 0.1 | Treat All | 0.404 | - | 0.596 | - | - | - |
| 0.2 | Machine Learning Algorithm | 0.241 | 0.496 | 0.093 | - | 0.161 | 0.161 |
| 0.2 | Genomic Classifier | 0.293 | 0.531 | 0.058 | - | 0.109 | 0.109 |
| 0.2 | Biopsy All | 0.284 | 0.502 | 0.056 | - | 0.095 | 0.095 |
| 0.2 | Treat All | 0.404 | - | 0.596 | - | - | - |
| 0.3 | Machine Learning Algorithm | 0.254 | 0.488 | 0.098 | - | 0.146 | 0.146 |
| 0.3 | Biopsy All | 0.284 | 0.502 | 0.056 | - | 0.095 | 0.095 |
| 0.3 | Genomic Classifier | 0.302 | 0.522 | 0.063 | - | 0.100 | 0.100 |
| 0.3 | Treat All | 0.404 | - | 0.596 | - | - | - |
| 0.4 | Machine Learning Algorithm | 0.267 | 0.480 | 0.103 | - | 0.132 | 0.132 |
| 0.4 | Biopsy All | 0.284 | 0.502 | 0.056 | - | 0.095 | 0.095 |
| 0.4 | Genomic Classifier | 0.311 | 0.514 | 0.068 | - | 0.090 | 0.090 |
| 0.4 | Treat All | 0.404 | - | 0.596 | - | - | - |
| 0.5 | Biopsy All | 0.284 | 0.502 | 0.056 | - | 0.095 | 0.095 |
| 0.5 | Machine Learning Algorithm | 0.281 | 0.472 | 0.107 | - | 0.117 | 0.117 |
| 0.5 | Genomic Classifier | 0.320 | 0.505 | 0.073 | - | 0.080 | 0.080 |
| 0.5 | Treat All | 0.404 | - | 0.596 | - | - | - |
| 0.6 | Biopsy All | 0.284 | 0.502 | 0.056 | - | 0.095 | 0.095 |
| 0.6 | Machine Learning Algorithm | 0.294 | 0.464 | 0.112 | - | 0.103 | 0.103 |
| 0.6 | Genomic Classifier | 0.329 | 0.496 | 0.078 | - | 0.070 | 0.070 |
| 0.6 | Treat All | 0.404 | - | 0.596 | - | - | - |
| 0.7 | Biopsy All | 0.284 | 0.502 | 0.056 | - | 0.095 | 0.095 |
| 0.7 | Machine Learning Algorithm | 0.307 | 0.456 | 0.117 | - | 0.088 | 0.088 |
| 0.7 | Genomic Classifier | 0.338 | 0.488 | 0.084 | - | 0.060 | 0.060 |
| 0.7 | Treat All | 0.404 | - | 0.596 | - | - | - |
| 0.8 | Biopsy All | 0.284 | 0.502 | 0.056 | - | 0.095 | 0.095 |
| 0.8 | Machine Learning Algorithm | 0.321 | 0.448 | 0.122 | - | 0.074 | 0.074 |
| 0.8 | Genomic Classifier | 0.347 | 0.479 | 0.089 | - | 0.050 | 0.050 |
| 0.8 | Treat All | 0.404 | - | 0.596 | - | - | - |
| 0.9 | Biopsy All | 0.284 | 0.502 | 0.056 | - | 0.095 | 0.095 |
| 0.9 | Machine Learning Algorithm | 0.334 | 0.440 | 0.127 | - | 0.059 | 0.059 |
| 0.9 | Genomic Classifier | 0.356 | 0.471 | 0.094 | - | 0.040 | 0.040 |
| 0.9 | Treat All | 0.404 | - | 0.596 | - | - | - |
| 1 | Biopsy All | 0.284 | 0.502 | 0.056 | - | 0.095 | 0.095 |
| 1 | Machine Learning Algorithm | 0.347 | 0.432 | 0.131 | - | 0.044 | 0.044 |
| 1 | Genomic Classifier | 0.365 | 0.462 | 0.099 | - | 0.030 | 0.030 |
| 1 | Treat All | 0.404 | - | 0.596 | - | - | - |

Table 4: Biopsy Utilization Rate by Algorithm Referral Rate

| **Probability of Algorithm Referral** | **Strategy** | **Biopsy Utilization** |
| --- | --- | --- |
| 0 | Machine Learning Algorithm | - |
| 0 | Genomic Classifier | - |
| 0 | Biopsy All | 1.000 |
| 0 | Treat All | - |
| 0.1 | Machine Learning Algorithm | 0.070 |
| 0.1 | Genomic Classifier | 0.068 |
| 0.1 | Biopsy All | 1.000 |
| 0.1 | Treat All | - |
| 0.2 | Machine Learning Algorithm | 0.140 |
| 0.2 | Genomic Classifier | 0.136 |
| 0.2 | Biopsy All | 1.000 |
| 0.2 | Treat All | - |
| 0.3 | Machine Learning Algorithm | 0.211 |
| 0.3 | Biopsy All | 1.000 |
| 0.3 | Genomic Classifier | 0.203 |
| 0.3 | Treat All | - |
| 0.4 | Machine Learning Algorithm | 0.281 |
| 0.4 | Biopsy All | 1.000 |
| 0.4 | Genomic Classifier | 0.271 |
| 0.4 | Treat All | - |
| 0.5 | Biopsy All | 1.000 |
| 0.5 | Machine Learning Algorithm | 0.351 |
| 0.5 | Genomic Classifier | 0.339 |
| 0.5 | Treat All | - |
| 0.6 | Biopsy All | 1.000 |
| 0.6 | Machine Learning Algorithm | 0.421 |
| 0.6 | Genomic Classifier | 0.407 |
| 0.6 | Treat All | - |
| 0.7 | Biopsy All | 1.000 |
| 0.7 | Machine Learning Algorithm | 0.492 |
| 0.7 | Genomic Classifier | 0.474 |
| 0.7 | Treat All | - |
| 0.8 | Biopsy All | 1.000 |
| 0.8 | Machine Learning Algorithm | 0.562 |
| 0.8 | Genomic Classifier | 0.542 |
| 0.8 | Treat All | - |
| 0.9 | Biopsy All | 1.000 |
| 0.9 | Machine Learning Algorithm | 0.632 |
| 0.9 | Genomic Classifier | 0.610 |
| 0.9 | Treat All | - |
| 1 | Biopsy All | 1.000 |
| 1 | Machine Learning Algorithm | 0.702 |
| 1 | Genomic Classifier | 0.678 |
| 1 | Treat All | - |

**Appendix 7: Sensitivity Analyses on the Sensitivity and Specificity of Supplemental Diagnostics**

Table 1: Sensitivity of the Machine Learning Algorithm

| **Algorithm Sensitivity** | **Strategy** | **Costs ($)** | **Incremental Cost ($)** | **Effectiveness** | **Incremental Effectiveness** | **ICER ($/QALY)** | **NMB, WTP $50K per QALY ($)** | **NMB, WTP $100K per QALY ($)** | **NMB, WTP $150K per QALY ($)** | **NMB, WTP $200K per QALY ($)** | **NMB ($), WTP $200K per QALY ($)** | **Dominance** |
| --- | --- | --- | --- | --- | --- | --- | --- | --- | --- | --- | --- | --- |
| 0.41 | Biopsy All | 334,037 | - | 3.62 | - | - | -152,801 | 28,434 | 209,670 | 390,905 | 572,141 |  |
| 0.41 | Machine Learning Algorithm | 367,534 | 33,498 | 3.73 | 0.11 | 313,725 | -180,960 | 5,614 | 192,189 | 378,763 | 565,337 |  |
| 0.41 | Genomic Classifier | 389,009 | 21,475 | 3.77 | 0.04 | 584,505 | -200,598 | -12,187 | 176,225 | 364,636 | 553,047 |  |
| 0.41 | Treat All | 711,579 | 322,570 | 3.86 | 0.1 | 3,370,788 | -518,383 | -325,187 | -131,991 | 61,205 | 254,401 |  |
| 0.46 | Biopsy All | 334,037 | - | 3.62 | - | - | -152,801 | 28,434 | 209,670 | 390,905 | 572,141 |  |
| 0.46 | Machine Learning Algorithm | 372,976 | 38,939 | 3.74 | 0.11 | 341,086 | -186,032 | 911 | 187,855 | 374,798 | 561,742 |  |
| 0.46 | Genomic Classifier | 389,009 | 16,033 | 3.77 | 0.03 | 546,247 | -200,598 | -12,187 | 176,225 | 364,636 | 553,047 |  |
| 0.46 | Treat All | 711,579 | 322,570 | 3.86 | 0.1 | 3,370,788 | -518,383 | -325,187 | -131,991 | 61,205 | 254,401 |  |
| 0.51 | Biopsy All | 334,037 | - | 3.62 | - | - | -152,801 | 28,434 | 209,670 | 390,905 | 572,141 |  |
| 0.51 | Machine Learning Algorithm | 378,417 | 44,381 | 3.75 | 0.12 | 365,120 | -191,104 | -3,791 | 183,522 | 370,835 | 558,148 |  |
| 0.51 | Genomic Classifier | 389,009 | 10,592 | 3.77 | 0.02 | 482,250 | -200,598 | -12,187 | 176,225 | 364,636 | 553,047 |  |
| 0.51 | Treat All | 711,579 | 322,570 | 3.86 | 0.1 | 3,370,788 | -518,383 | -325,187 | -131,991 | 61,205 | 254,401 |  |
| 0.56 | Biopsy All | 334,037 | - | 3.62 | - | - | -152,801 | 28,434 | 209,670 | 390,905 | 572,141 |  |
| 0.56 | Machine Learning Algorithm | 383,859 | 49,822 | 3.75 | 0.13 | 386,400 | -196,177 | -8,494 | 179,188 | 366,871 | 554,553 | Extended |
| 0.56 | Genomic Classifier | 389,009 | 54,972 | 3.77 | 0.14 | 383,046 | -200,598 | -12,187 | 176,225 | 364,636 | 553,047 |  |
| 0.56 | Treat All | 711,579 | 322,570 | 3.86 | 0.1 | 3,370,788 | -518,383 | -325,187 | -131,991 | 61,205 | 254,401 |  |
| 0.61 | Biopsy All | 334,037 | - | 3.62 | - | - | -152,801 | 28,434 | 209,670 | 390,905 | 572,141 |  |
| 0.61 | Genomic Classifier | 389,009 | 54,972 | 3.77 | 0.14 | 383,046 | -200,598 | -12,187 | 176,225 | 364,636 | 553,047 |  |
| 0.61 | Machine Learning Algorithm | 389,300 | 291 | 3.76 | -0.01 | -40,514 | -201,248 | -13,196 | 174,855 | 362,907 | 550,959 | Absolute |
| 0.61 | Treat All | 711,579 | 322,570 | 3.86 | 0.1 | 3,370,788 | -518,383 | -325,187 | -131,991 | 61,205 | 254,401 |  |
| 0.65 | Biopsy All | 334,037 | - | 3.62 | - | - | -152,801 | 28,434 | 209,670 | 390,905 | 572,141 |  |
| 0.65 | Genomic Classifier | 389,009 | 54,972 | 3.77 | 0.14 | 383,046 | -200,598 | -12,187 | 176,225 | 364,636 | 553,047 |  |
| 0.65 | Machine Learning Algorithm | 393,654 | 4,644 | 3.77 | 0 | -3,640,205 | -205,306 | -16,959 | 171,389 | 359,736 | 548,084 | Absolute |
| 0.65 | Treat All | 711,579 | 322,570 | 3.86 | 0.1 | 3,370,788 | -518,383 | -325,187 | -131,991 | 61,205 | 254,401 |  |

Table 2: Specificity of the Machine Learning Algorithm

| **Algorithm Specificity** | **Strategy** | **Costs ($)** | **Incremental Cost ($)** | **Effectiveness** | **Incremental Effectiveness** | **ICER ($/QALY)** | **NMB, WTP $50K per QALY ($)** | **NMB, WTP $100K per QALY ($)** | **NMB, WTP $150K per QALY ($)** | **NMB, WTP $200K per QALY ($)** | **NMB ($), WTP $200K per QALY ($)** | **Dominance** |
| --- | --- | --- | --- | --- | --- | --- | --- | --- | --- | --- | --- | --- |
| 0.77 | Biopsy All | 334,037 | - | 3.62 | - | - | -152,801 | 28,434 | 209,670 | 390,905 | 572,141 |  |
| 0.77 | Genomic Classifier | 389,009 | 54,972 | 3.77 | 0.14 | 383,046 | -200,598 | -12,187 | 176,225 | 364,636 | 553,047 |  |
| 0.77 | Machine Learning Algorithm | 410,402 | 21,393 | 3.75 | -0.01 | -1,498,940 | -222,704 | -35,007 | 152,691 | 340,388 | 528,086 | Absolute |
| 0.77 | Treat All | 711,579 | 322,570 | 3.86 | 0.1 | 3,370,788 | -518,383 | -325,187 | -131,991 | 61,205 | 254,401 |  |
| 0.82 | Biopsy All | 334,037 | - | 3.62 | - | - | -152,801 | 28,434 | 209,670 | 390,905 | 572,141 |  |
| 0.82 | Genomic Classifier | 389,009 | 54,972 | 3.77 | 0.14 | 383,046 | -200,598 | -12,187 | 176,225 | 364,636 | 553,047 |  |
| 0.82 | Machine Learning Algorithm | 393,842 | 4,833 | 3.75 | -0.02 | -285,920 | -206,276 | -18,710 | 168,856 | 356,422 | 543,988 | Absolute |
| 0.82 | Treat All | 711,579 | 322,570 | 3.86 | 0.1 | 3,370,788 | -518,383 | -325,187 | -131,991 | 61,205 | 254,401 |  |
| 0.87 | Biopsy All | 334,037 | - | 3.62 | - | - | -152,801 | 28,434 | 209,670 | 390,905 | 572,141 |  |
| 0.87 | Machine Learning Algorithm | 377,282 | 43,245 | 3.75 | 0.12 | 348,808 | -189,847 | -2,413 | 185,022 | 372,456 | 559,891 |  |
| 0.87 | Genomic Classifier | 389,009 | 11,727 | 3.77 | 0.02 | 600,346 | -200,598 | -12,187 | 176,225 | 364,636 | 553,047 |  |
| 0.87 | Treat All | 711,579 | 322,570 | 3.86 | 0.1 | 3,370,788 | -518,383 | -325,187 | -131,991 | 61,205 | 254,401 |  |
| 0.92 | Biopsy All | 334,037 | - | 3.62 | - | - | -152,801 | 28,434 | 209,670 | 390,905 | 572,141 |  |
| 0.92 | Machine Learning Algorithm | 360,722 | 26,685 | 3.75 | 0.12 | 219,903 | -173,419 | 13,884 | 201,187 | 388,490 | 575,793 |  |
| 0.92 | Genomic Classifier | 389,009 | 28,287 | 3.77 | 0.02 | 1,276,213 | -200,598 | -12,187 | 176,225 | 364,636 | 553,047 |  |
| 0.92 | Treat All | 711,579 | 322,570 | 3.86 | 0.1 | 3,370,788 | -518,383 | -325,187 | -131,991 | 61,205 | 254,401 |  |

Table 3: Sensitivity of the Genomic Classifier

| **Genomic Classifier Sensitivity** | **Strategy** | **Costs ($)** | **Incremental Cost ($)** | **Effectiveness** | **Incremental Effectiveness** | **ICER ($/QALY)** | **NMB, WTP $50K per QALY ($)** | **NMB, WTP $100K per QALY ($)** | **NMB, WTP $150K per QALY ($)** | **NMB, WTP $200K per QALY ($)** | **NMB ($),WTP $250K per QALY ($)** | **Dominance** |
| --- | --- | --- | --- | --- | --- | --- | --- | --- | --- | --- | --- | --- |
| 0.55 | Biopsy All | 334,037 | - | 3.62 | - | - | -152,801 | 28,434 | 209,670 | 390,905 | 572,141 |  |
| 0.55 | Genomic Classifier | 374,861 | 40,825 | 3.75 | 0.12 | 328,425 | -187,410 | 40 | 187,491 | 374,941 | 562,392 |  |
| 0.55 | Machine Learning Algorithm | 380,594 | 5,733 | 3.75 | 0 | 28,403,261 | -193,133 | -5,672 | 181,788 | 369,249 | 556,710 | Extended |
| 0.55 | Treat All | 711,579 | 336,718 | 3.86 | 0.11 | 2,930,389 | -518,383 | -325,187 | -131,991 | 61,205 | 254,401 |  |
| 0.6 | Biopsy All | 334,037 | - | 3.62 | - | - | -152,801 | 28,434 | 209,670 | 390,905 | 572,141 |  |
| 0.6 | Genomic Classifier | 380,303 | 46,266 | 3.76 | 0.13 | 351,319 | -192,483 | -4,663 | 183,158 | 370,978 | 558,798 |  |
| 0.6 | Machine Learning Algorithm | 380,594 | 291 | 3.75 | -0.01 | -40,514 | -193,133 | -5,672 | 181,788 | 369,249 | 556,710 | Absolute |
| 0.6 | Treat All | 711,579 | 331,276 | 3.86 | 0.11 | 3,081,150 | -518,383 | -325,187 | -131,991 | 61,205 | 254,401 |  |
| 0.65 | Biopsy All | 334,037 | - | 3.62 | - | - | -152,801 | 28,434 | 209,670 | 390,905 | 572,141 |  |
| 0.65 | Machine Learning Algorithm | 380,594 | 46,557 | 3.75 | 0.12 | 373,936 | -193,133 | -5,672 | 181,788 | 369,249 | 556,710 | Extended |
| 0.65 | Genomic Classifier | 385,744 | 51,707 | 3.76 | 0.14 | 371,780 | -197,554 | -9,365 | 178,825 | 367,014 | 555,204 |  |
| 0.65 | Treat All | 711,579 | 325,835 | 3.86 | 0.1 | 3,254,159 | -518,383 | -325,187 | -131,991 | 61,205 | 254,401 |  |
| 0.7 | Biopsy All | 334,037 | - | 3.62 | - | - | -152,801 | 28,434 | 209,670 | 390,905 | 572,141 |  |
| 0.7 | Machine Learning Algorithm | 380,594 | 46,557 | 3.75 | 0.12 | 373,936 | -193,133 | -5,672 | 181,788 | 369,249 | 556,710 |  |
| 0.7 | Genomic Classifier | 391,186 | 10,592 | 3.77 | 0.02 | 482,250 | -202,627 | -14,068 | 174,491 | 363,050 | 551,609 |  |
| 0.7 | Treat All | 711,579 | 320,393 | 3.86 | 0.09 | 3,454,735 | -518,383 | -325,187 | -131,991 | 61,205 | 254,401 |  |
| 0.73 | Biopsy All | 334,037 | - | 3.62 | - | - | 572,141 | 28,434 | 209,670 | 390,905 | 572,141 |  |
| 0.73 | Machine Learning Algorithm | 380,594 | 46,557 | 3.75 | 0.12 | 373,936 | 556,710 | -5,672 | 181,788 | 369,249 | 556,710 |  |
| 0.73 | Genomic Classifier | 394,451 | 13,857 | 3.78 | 0.03 | 524,947 | 549,452 | -16,890 | 171,891 | 360,671 | 549,452 |  |
| 0.73 | Treat All | 711,579 | 317,128 | 3.86 | 0.09 | 3,591,191 | 254,401 | -325,187 | -131,991 | 61,205 | 254,401 |  |

Table 4: Specificity of the Genomic Classifier

| **Genomic Classifier Specificity** | **Strategy** | **Costs ($)** | **Incremental Cost ($)** | **Effectiveness** | **Incremental Effectiveness** | **ICER ($/QALY)** | **NMB, WTP $50K per QALY ($)** | **NMB, WTP $100K per QALY ($)** | **NMB, WTP $150K per QALY ($)** | **NMB, WTP $200K per QALY ($)** | **NMB ($),WTP $200K per QALY ($)** | **Dominance** |
| --- | --- | --- | --- | --- | --- | --- | --- | --- | --- | --- | --- | --- |
| 0.81 | Biopsy All | 334,037 | - | 3.62 | - | - | -152,801 | 28,434 | 209,670 | 390,905 | 572,141 |  |
| 0.81 | Machine Learning Algorithm | 380,594 | 46,557 | 3.75 | 0.12 | 373,936 | -193,133 | -5,672 | 181,788 | 369,249 | 556,710 |  |
| 0.81 | Genomic Classifier | 425,442 | 44,848 | 3.77 | 0.02 | 1,808,656 | -236,741 | -48,040 | 140,660 | 329,361 | 518,062 |  |
| 0.81 | Treat All | 711,579 | 286,137 | 3.86 | 0.09 | 3,182,575 | -518,383 | -325,187 | -131,991 | 61,205 | 254,401 |  |
| 0.86 | Biopsy All | 334,037 | - | 3.62 | - | - | -152,801 | 28,434 | 209,670 | 390,905 | 572,141 |  |
| 0.86 | Machine Learning Algorithm | 380,594 | 46,557 | 3.75 | 0.12 | 373,936 | -193,133 | -5,672 | 181,788 | 369,249 | 556,710 |  |
| 0.86 | Genomic Classifier | 408,881 | 28,287 | 3.77 | 0.02 | 1,276,213 | -220,312 | -31,743 | 156,826 | 345,395 | 533,964 |  |
| 0.86 | Treat All | 711,579 | 302,698 | 3.86 | 0.09 | 3,271,045 | -518,383 | -325,187 | -131,991 | 61,205 | 254,401 |  |
| 0.91 | Biopsy All | 334,037 | - | 3.62 | - | - | -152,801 | 28,434 | 209,670 | 390,905 | 572,141 |  |
| 0.91 | Machine Learning Algorithm | 380,594 | 46,557 | 3.75 | 0.12 | 373,936 | -193,133 | -5,672 | 181,788 | 369,249 | 556,710 |  |
| 0.91 | Genomic Classifier | 392,321 | 11,727 | 3.77 | 0.02 | 600,346 | -203,884 | -15,446 | 172,991 | 361,429 | 549,866 |  |
| 0.91 | Treat All | 711,579 | 319,258 | 3.86 | 0.1 | 3,354,624 | -518,383 | -325,187 | -131,991 | 61,205 | 254,401 |  |
| 0.95 | Biopsy All | 334,037 | - | 3.62 | - | - | -152,801 | 28,434 | 209,670 | 390,905 | 572,141 |  |
| 0.95 | Genomic Classifier | 379,073 | 45,036 | 3.77 | 0.14 | 317,301 | -190,741 | -2,409 | 185,924 | 374,256 | 562,588 |  |
| 0.95 | Machine Learning Algorithm | 380,594 | 1,521 | 3.75 | -0.02 | -87,261 | -193,133 | -5,672 | 181,788 | 369,249 | 556,710 | Absolute |
| 0.95 | Treat All | 711,579 | 332,506 | 3.86 | 0.1 | 3,418,232 | -518,383 | -325,187 | -131,991 | 61,205 | 254,401 |  |

**Appendix 8: Sensitivity Analysis on the Prevalence of IPF**

| **IPF Prevalence** | **Strategy** | **Costs ($)** | **Incremental Cost ($)** | **Effectiveness** | **Incremental Effectiveness** | **ICER ($/QALY)** | **NMB, WTP $50K per QALY ($)** | **NMB, WTP $100K per QALY ($)** | **NMB, WTP $150K per QALY ($)** | **NMB, WTP $200K per QALY ($)** | **NMB ($),WTP $200K per QALY ($)** | **Dominance** |
| --- | --- | --- | --- | --- | --- | --- | --- | --- | --- | --- | --- | --- |
| 0.3 | Biopsy All | 294,332.28 | - | 3.6 | - | - | -114,501 | 65,330 | 245,161 | 424,993 | 604,824 |  |
| 0.3 | Machine Learning Algorithm | 344,058.52 | 49,726.24 | 3.71 | 0.11 | 441,416 | -158,595 | 26,869 | 212,333 | 397,797 | 583,261 | Extended |
| 0.3 | Genomic Classifier | 344,803.76 | 50,471.47 | 3.72 | 0.13 | 402,475 | -158,702 | 27,399 | 213,501 | 399,602 | 585,704 |  |
| 0.3 | Treat All | 711,579.00 | 366,775.24 | 3.81 | 0.09 | 3,987,965 | -520,879 | -330,179 | -139,479 | 51,221 | 241,921 |  |
| 0.35 | Biopsy All | 313,421.02 | - | 3.61 | - | - | -132,915 | 47,592 | 228,098 | 408,605 | 589,111 |  |
| 0.35 | Machine Learning Algorithm | 361,623.66 | 48,202.64 | 3.73 | 0.12 | 407,286 | -175,200 | 11,224 | 197,648 | 384,072 | 570,496 | Extended |
| 0.35 | Genomic Classifier | 366,056.38 | 52,635.36 | 3.74 | 0.13 | 392,479 | -178,845 | 8,367 | 195,579 | 382,791 | 570,003 |  |
| 0.35 | Treat All | 711,579.00 | 345,522.62 | 3.84 | 0.09 | 3,685,124 | -519,679 | -327,779 | -135,879 | 56,021 | 247,921 |  |
| 0.4 | Biopsy All | 332,509.75 | - | 3.62 | - | - | -151,328 | 29,853 | 211,035 | 392,216 | 573,398 |  |
| 0.4 | Machine Learning Algorithm | 379,188.80 | 46,679.05 | 3.75 | 0.12 | 376,292 | -191,805 | -4,421 | 182,963 | 370,347 | 557,731 |  |
| 0.4 | Genomic Classifier | 387,309.00 | 8,120.20 | 3.77 | 0.02 | 432,678 | -198,987 | -10,664 | 177,658 | 365,981 | 554,303 |  |
| 0.4 | Treat All | 711,579.00 | 324,270.00 | 3.86 | 0.1 | 3,393,636 | -518,479 | -325,379 | -132,279 | 60,821 | 253,921 |  |
| 0.45 | Biopsy All | 351,598.48 | - | 3.64 | - | - | -169,742 | 12,115 | 193,972 | 375,828 | 557,685 |  |
| 0.45 | Machine Learning Algorithm | 396,753.94 | 45,155.46 | 3.77 | 0.13 | 348,021 | -208,410 | -20,066 | 168,278 | 356,622 | 544,966 |  |
| 0.45 | Genomic Classifier | 408,561.62 | 11,807.68 | 3.79 | 0.02 | 542,250 | -219,129 | -29,696 | 159,737 | 349,170 | 538,603 |  |
| 0.45 | Treat All | 711,579.00 | 303,017.38 | 3.89 | 0.1 | 3,112,873 | -517,279 | -322,979 | -128,679 | 65,621 | 259,921 |  |
| 0.5 | Biopsy All | 370,687.21 | - | 3.65 | - | - | -188,156 | -5,624 | 176,908 | 359,439 | 541,971 |  |
| 0.5 | Machine Learning Algorithm | 414,319.08 | 43,631.86 | 3.79 | 0.14 | 322,129 | -225,015 | -35,711 | 153,594 | 342,898 | 532,202 |  |
| 0.5 | Genomic Classifier | 429,814.24 | 15,495.16 | 3.81 | 0.02 | 625,223 | -239,271 | -48,728 | 141,816 | 332,359 | 522,902 |  |
| 0.5 | Treat All | 711,579.00 | 281,764.76 | 3.91 | 0.1 | 2,842,255 | -516,079 | -320,579 | -125,079 | 70,421 | 265,921 |  |
| 0.55 | Biopsy All | 389,775.95 | - | 3.66 | - | - | -206,569 | -23,362 | 159,844 | 343,051 | 526,258 |  |
| 0.55 | Machine Learning Algorithm | 431,884.22 | 42,108.27 | 3.81 | 0.14 | 298,328 | -241,620 | -51,356 | 138,909 | 329,173 | 519,437 |  |
| 0.55 | Genomic Classifier | 451,066.86 | 19,182.64 | 3.83 | 0.03 | 690,234 | -259,413 | -67,759 | 123,894 | 315,548 | 507,202 |  |
| 0.55 | Treat All | 711,579.00 | 260,512.14 | 3.93 | 0.1 | 2,581,241 | -514,879 | -318,179 | -121,479 | 75,221 | 271,921 |  |
| 0.6 | Biopsy All | 408,864.68 | - | 3.68 | - | - | -224,983 | -41,101 | 142,781 | 326,663 | 510,545 |  |
| 0.6 | Machine Learning Algorithm | 449,449.36 | 40,584.68 | 3.82 | 0.15 | 276,374 | -258,225 | -67,001 | 124,223 | 315,448 | 506,672 |  |
| 0.6 | Genomic Classifier | 472,319.48 | 22,870.13 | 3.86 | 0.03 | 742,547 | -279,555 | -86,791 | 105,973 | 298,737 | 491,501 |  |
| 0.6 | Treat All | 711,579.00 | 239,259.52 | 3.96 | 0.1 | 2,329,328 | -513,679 | -315,779 | -117,879 | 80,021 | 277,921 |  |
